# Developing the African national health research systems barometer

**DOI:** 10.1186/s12961-016-0121-4

**Published:** 2016-07-22

**Authors:** Joses Muthuri Kirigia, Martin Okechukwu Ota, Flavia Senkubuge, Charles Shey Wiysonge, Bongani M. Mayosi

**Affiliations:** Community-Based Research, Innovation and Sustainable Development Organisation (CRSDO), P.O. Box 6994, 00100, GPO Nairobi, Kenya; Health Systems and Services Cluster, World Health Organization Regional Office for Africa, Brazzaville, Congo; Health Policy and Management Department, School of Health Systems and Public Health University of Pretoria, Pretoria, South Africa; Centre for Evidence-based Health Care, Stellenbosch University, Cape Town, South Africa; Department of Medicine, Groote Schuur Hospital and University of Cape Town, Cape Town, South Africa

**Keywords:** National health research systems performance, Research for health governance, Research production and utilization, Research financing, Research coordination

## Abstract

**Background:**

A functional national health research system (NHRS) is crucial in strengthening a country’s health system to promote, restore and maintain the health status of its population. Progress towards the goal of universal health coverage in the post-2015 sustainable development agenda will be difficult for African countries without strengthening of their NHRS to yield the required evidence for decision-making. This study aims to develop a barometer to facilitate monitoring of the development and performance of NHRSs in the African Region of WHO.

**Methods:**

The African national health research systems barometer algorithm was developed in response to a recommendation of the African Advisory Committee for Health Research and Development of WHO. Survey data collected from all the 47 Member States in the WHO African Region using a questionnaire were entered into an Excel spreadsheet and analysed. The barometer scores for each country were calculated and the performance interpreted according to a set of values ranging from 0% to 100%.

**Results:**

The overall NHRS barometer score for the African Region was 42%, which is below the average of 50%. Among the 47 countries, the average NHRS performance was less than 20% in 10 countries, 20–40% in 11 countries, 41–60% in 16 countries, 61–80% in nine countries, and over 80% in one country. The performance of NHRSs in 30 (64%) countries was below 50%.

**Conclusion:**

An African NHRS barometer with four functions and 17 sub-functions was developed to identify the gaps in and facilitate monitoring of NHRS development and performance. The NHRS scores for the individual sub-functions can guide policymakers to locate sources of poor performance and to design interventions to address them.

**Electronic supplementary material:**

The online version of this article (doi:10.1186/s12961-016-0121-4) contains supplementary material, which is available to authorized users.

## Background

The African Region of WHO bears a double burden of communicable and non-communicable diseases, and many of its countries have not achieved the health Millennium Development Goals (MDGs) [1]. Health research is critical in providing evidence-based solutions for the much-needed improvement in health and development. A functional national health research system (NHRS) is vital for optimising research generation, dissemination and utilisation in addressing the health needs of the population.

An NHRS is defined as the people, institutions and activities whose primary purpose is to generate and promote the utilisation of high quality scientific knowledge to promote, restore and/or maintain the health of a population [2]. Its goals are to advance scientific knowledge and promote its utilisation in strengthening national health systems to be responsive, to provide social and financial risk protection, to improve efficiency of services, and ultimately to improve the health of the population. An NHRS has four functions: governance/stewardship, developing and sustaining resources, financing, and producing and using research for health (R4H).

Governance is an overarching function of the government to ensure effective oversight, coalition building, system design, accountability, and regulation of all R4H taking place in both the public and private sectors. The indicators for R4H governance include availability and implementation of a national R4H policy and strategic plan, a R4H agenda, a R4H legislation or law (including ethical standards and guidelines), a R4H focal point, and a functional ethical review committee to protect the dignity, integrity and safety of research participants.

The developing and sustaining R4H resources function covers building or strengthening and sustaining of human resources for biomedical, bioscience, epidemiology, public health, social science and health system research, physical infrastructure, and institutional and systemic capacities to manage knowledge [3]. In this paper, the parameters for this function include the number of universities of health sciences conducting research, the number of national health research institutes or the existence of a council, the presence of a health research programme in the ministry of health, and the number of researchers and support staff in a R4H programme.

The function of producing and using R4H entails the conduct, dissemination and application of R4H in advocacy, policy and planning, systems strengthening, programme implementation, development of new products and tools, monitoring and evaluation, etc. [3, 4]. The quantity of R4H produced in each country can be determined through the analysis of biomedical papers indexed in electronic databases such as Medline, PubMed, PsycInfo or Web of Science [5, 6].

The existence of a knowledge platform that is designed for translating, synthesising and communicating research to inform health policy and practice could be used as a proxy for the use of research findings. In this paper, the parameters for the production and use of the research function include the existence of a R4H programme action plan, a knowledge translation platform and a national R4H management forum, and the number of R4H articles published per person.

Financing of R4H refers to the estimation of recurrent and capital cost of R4H; mobilisation/collection of R4H funds from individuals, businesses, the government and donors; accumulation and management of R4H funds; allocation of funds to individuals, institutions and networks within NHRSs that govern R4H, create R4H inputs, produce R4H, and monitor and evaluate R4H; and tracking of expenditures on R4H. The parameters for the financing function include the ministry of health’s budget line for R4H and the government’s allocation of 2% of the national health budget to R4H, per the Algiers Declaration of Health Ministers in 2008 [7].

As the world moves towards the post-2015 sustainable development agenda [8], which includes provision of universal health coverage, people will need equitable access to health systems that can deliver high quality services where and when they are needed. The WHO World Health Report 2013 stated that universal health coverage, with full access to high quality services for prevention, treatment and financial risk protection, cannot be achieved without evidence from scientific research [2]. It was also unequivocal that every country needed an effective NHRS to set research priorities, develop research capacity, define norms and standards for research, and translate evidence into practice. This is particularly essential for the African Region, for research to identify the ways of increasing the utilisation of available interventions as well as the new tools to tackle emerging and re-emerging diseases.

The 28th session of the WHO African Advisory Committee for Health Research and Development in 2014 recommended the development of a barometer to help in assessing and tracking of the performance of NHRSs in countries of the WHO African Region [6]. This paper describes the process to develop such a barometer.

## Methods

### Steps in developing the NHRS barometer

#### Step 1: Delineate the NHRS functions

The proposed barometer will have four functions, derived from the standard NHRS conceptual framework [2]. These are governance of R4H, developing and sustaining resources for R4H, producing and using R4H, and financing R4H.

#### Step 2: Delineate the sub-functions under each NHRS function

In this paper, governance has six sub-functions, whereas developing and sustaining resources has five, producing and using research has four, and financing has two, making a total of 17 sub-functions (Box 1) for which scores were estimated. The choice of the sub-functions was made from a culmination of research on NHRS covering over a decade [4–6, 9–17]. The 17 sub-functions were critically reviewed and adapted by the African Advisory Committee on Health Research and Development [3] as proximate indicators for use in monitoring the implementation of the African regional strategy on research for health and development of NHRSs in the Region [17].

**Table 1:** Regional health research system barometer scores

Health research system barometer parameters	Regional barometer score
A. Governance of research for health	
1. Regional health research policy index (RHRPI)	0.49
2. Regional health research law index (RHRLI)	0.40
3. Regional strategic health research plan index (RSHRPI)	0.47
4. Regional ethical review committee index (RERCI)	0.91
5. Regional health research priority list index (RHRPLI)	0.53
6. Regional health research focal point index (RHRFPI)	0.83
Average score for the governance of R4H	*0.61*
B. Developing and sustaining resources for R4H	
7. Regional universities with faculties of health sciences/medicine (RUFHSI)	0.05
8. Regional health research institutes or council (RHRCI)	0.55
9. Regional R4H programme (RHRPRI)	0.51
10. Regional R4H programme staff density index (RHRHRI)	0.0006
11. Regional NGOs R4H index (RNGOI)	0.64
Average score for developing and sustaining resources for R4H	*0.35*
C. Producing and using research	
12. Regional R4H programme action plan index (RHRPAI)	0.34
13. Regional knowledge translation platform index (RKTPI)	0.32
14. Regional health research management forum index (RHRMFI)	0.51
15. Regional R4H publications per 100,000 population index (RPPCI)	0.10
Average score for producing and using research	*0.32*
D. Financing of R4H	
16. Regional budget line for R4H index (RBLHRI)	0.47
17. Regional government spending on R4H index (RHRBI)	0.06
*Average score for financing of R4H*	*0.27*
Regional health research systems barometer (RHRSB) average score	*0.42*

**Table 2:** African countries’ national research for health system barometer scores in 2014 by economic group

Group 1: High income and upper middle income (*n* = 9)	
Country	Score
Algeria	59
Angola	47
Botswana	55
Equatorial Guinea	13
Gabon	19
Mauritius	19
Namibia	24
Seychelles	18
South Africa	79
*Average*	*37*
*Median*	*24*
*STDEV*	*24*
*Max*	*79*
*Min*	*13*
Group 2: Lower middle income (*n* = 13)	
*Countries*	*Score*
Cameroun	36
Cape Verde	50
Congo	24
Cote D’Ivoire	36
Ghana	48
Kenya	42
Lesotho	47
Mauritania	30
Nigeria	42
Sao Tome and Principe	6
Senegal	71
Swaziland	54
Zambia	65
*Average*	*42*
*Median*	*42*
*STDEV*	*17*
*Max*	*71*
*Min*	*6*
Group 3: Low income (*n* = 25)	
Countries	Score
Burkina Faso	65
Burundi	35
Benin	54
Chad	12
CAR	30
Comoros	18
DRC	35
Eritrea	42
Ethiopia	65
Gambia	43
Guinea	53
Guinea-Bissau	30
Liberia	36
Madagascar	42
Malawi	48
Mali	59
Mozambique	30
Niger	65
Rwanda	81
Sierra Leone	18
South Sudan	12
Tanzania	77
Togo	18
Uganda	72
Zimbabwe	65
*Average*	*44*
*Median*	*42*
*STDEV*	*21*
*Max*	*81*
*Min*	*12*

**Box 1:** National health research systems sub-functions

Functions and sub-functions	
*A. Governance of research for health (R4H)*	
1. National policy on research for health	
2. Strategic plan on research for health	
3. Law governing research	
4. National research for health priority list/agenda	
5. National ethics review committee	
6. National health research focal point	
*B. Developing and sustaining resources for R4H*	
7. University colleges of health sciences conducting research	
8. National health research institutes or council	
9. Health research programme at MOH	
10. Number of researchers in a R4H programme	
11. Non-governmental organisations conducting R4H	
*C. Producing and using research*	
12. R4H programme action plan	
13. Existence of knowledge translation platform	
14. Existence of health research management forum	
15. R4H peer-reviewed publications per 100,000 population	
*D. Financing of R4H*	
16. Existence of a budget line in the health budget for research for health	
17. Progress towards the target of allocating 2% of national health budget on R4H	

#### Step 3: Collect data on each sub-function

A shortened version of the questionnaire used by Kirigia and Wambebe in 2003 [9] and Mbondji et al. [16] in 2009 was used in the 2014 survey [18] to collect data used in estimating the barometer scores for all the 47 Member States in the WHO African Region.

The R4H questionnaires used in 2003, 2009 and 2014 had the same 10 broad components, i.e. health research policy, health research legislation, strategic health research plan, research coordination mechanisms, health research programme, research institutes, national universities, health research financing and budget, non-governmental organizations involved in health research, and actions needed to strengthen health research capacity. However, six components in the 2014 short version had fewer questions compared to the 2003/2009 versions. The Health Research Policy component had three questions instead of 11 – the contents of health research policy, complementarity with national health policies, stakeholder participation, and technical support needs from WHO were omitted. The Strategic Health Research Plan component had three instead six questions – period covered and need for WHO technical support were omitted. The Research Coordination Mechanisms component had eight instead of 15 questions – those on the national health research management forum, ethical review committee, Scientific Review Committee, Institutional Review Committee, and Hospital Ethics Review Committee terms of reference and frequency of meetings was omitted. The National Health Research Institutes (NHRI) component had five instead of 15 questions – those enquiring when it was started, under which ministry, information and communication technologies connectivity, and regarding WHO Collaborating Centres were omitted. Health Research Financing and Budget had four instead of seven questions – queries on total government budget and total spending on research in general from all sources were missing. The rationale for using the short version was to reduce the amount of time needed for completion and to increase the response rate. The majority of questions omitted were those that entailed qualitative subjective judgements of respondents. In the NHRS assessments conducted in the region in 2003 and 2009 using the long questionnaire, only 10 and 44 countries, respectively, responded. We believe that the use of the short version of the questionnaire contributed to having completed questionnaires from all the 47 Member States in the WHO African region. We have no reason to believe that the omitted questions influenced the values of the indices for sub-functions and the overall composite index. Since the questions in the 2014 short version questionnaire are those used in the previous two surveys, authors did not think there was need for pilot testing or pretesting.

The questionnaire was sent by email to all the 47 Member States in the WHO African Region through the WHO country representatives. It was accompanied with a covering memo from the WHO Regional Director underscoring the urgency of the survey and asking the country representatives to personally facilitate and follow-up on the survey. The national health research focal person in each of the countries, irrespective of the government ministry hosting them, had the primary responsibility for completing the questionnaire. The WHO country office national professional officer in charge of research hand-delivered the questionnaire to the national focal point, provided a briefing on the survey, went through the questionnaire with a national focal point and closely monitored the progress on the completion of the questionnaire. All the 47 countries completed and returned the questionnaire to the Regional Office for Africa. Excerpts of the questions related to the sub-functions are contained in Box 2. The methodological details on how the survey was conducted are contained in an article published in *Health Research Policy and Systems* [18].

**Box 2:** Questions related to national health research systems sub-functions

Functions and sub-functions	
*A. Governance of research for health*	
1. National policy on research for health: Does the country have a valid official health research policy? (Yes or No)	
2. Strategic plan on research for health: Does the country have a strategic health research plan? (Yes or No)	
3. Law governing research: Does the country have a law relating to health research? (Yes or No)	
4. National research for health priority list/agenda: Does the country have a health research priority list? (Yes or No)	
5. National ethics review committee: Does the country have a functional ethical review committee? (Yes or No)	
6. National health research focal point: Is there a national health research focal point in the country? (Yes or No)	
*B. Developing and sustaining resources for R4H*	
1. University colleges of health sciences conducting research: Does the country have faculties of health sciences that conduct research for health? (Yes or No)	
2. National health research institute(s) or council: Does the country have a national health research institute(s) or council? (Yes or No)	
3. Health research programme at MoH: Does the country have a health research programme? (Yes or No)	
4. Number of technical and support staff in a R4H programme: How many technical and support staff are there in the programme? (Yes or No)	
5. Non-governmental organisations conducting R4H: Are there any NGOs in the country that undertake health research? (Yes or No)	
*C. Producing and using research*	
1. R4H programme action plan: Does the R4H programme have a plan of action?	
2. Existence of knowledge translation platform: Does the country have a platform for translating, synthesising and communicating research to inform health policy and practice? (Yes or No)	
3. R4H peer-reviewed publications per 100,000 population*	
4. National R4H management forum: Is there a functional national health research forum? (Yes or No)	
*D. Financing of R4H*	
1. Existence of a budget line in the health budget for research for health: Is there a budget line for research for health in the Ministry of Health budget document? (Yes or No)	
2. Progress towards the target of allocating 2% of the national health budget to R4H: (a) How much money (in local currency) did the Ministry of Health allocate to research in the last financial year (2012/2013)? (b) What was the Ministry of Health’s overall budget (in local currency) in the last financial year (2012/2013)?	

*Data for this question were missing from the survey, so they were obtained from other sources [17]

All the data used in this study were collected using the questionnaire mentioned above. The questionnaire did not, however, capture data on the total peer-reviewed R4H articles per country in the Region, as most of the countries did not have a repository with that information. Therefore, those data were obtained from a bibliometric study published by some of the authors of this article [19].

#### Step 4: Score the sub-functions

Taking into account the information from the completed questionnaires on each country’s NHRS (step 3), we assessed the dichotomous/categorical sub-functions and allocated them a percentage score ranging from 0% for non-existence to 100% for existence. For example, if with regards to national policy on research for health, a respondent answered ‘yes’ to the question ‘Does the country have a valid official health research policy?’, that country received a score of 100% for that sub-function, while a country that had ‘no’ for an answer received 0%. This exercise was undertaken for all the questions with a ‘yes’ or a ‘no’ answer.

For sub-functions with continuous value answers, the score was the actual value. For example, for the number of technical and support staff sub-function question ‘How many technical and support staff are there in the programme?’ the actual value was the number of staff per 100,000 population, that is staff density. This value was obtained by dividing the total number of staff by the population and multiplying the outcome by 100,000. Similarly, the actual value for R4H peer-reviewed publications per 100,000 population was estimated through the division of the total number of R4H articles published by the population and multiplying the outcome by 100,000.

Another example of a sub-function with a continuous value is the number of universities with faculties of health sciences (or medicine), where the actual value was the number of universities with faculties of health sciences per 1 million population. This value was calculated by dividing the number of national universities with faculties of health sciences or medicine by the country’s population and multiplying the outcome by 1 million.

Concerning the sub-function entitled ‘progress towards the target of allocating 2% of the national health budget on R4H’, the score was the ratio of the money budgeted by the Ministry of Health (MOH) for research in the last financial year (2012/2013) to the overall MOH budget for that year multiplied by 100%.

#### Step 5: Calculate sub-function indices

All the indices for the 17 individual sub-functions were calculated using the following general formula:

$$ Sub\  function\  index=\left(\frac{Actual\  xi\  score - Minimum\  xi\  Score}{Maximum\  xi\  score- Minimum\  xi\  score}\right) $$

where x_i_ is the i^th^ sub-function, such as the existence of a national policy on research for health (R4HP), a strategic plan on research for health, a national research for health priority list/agenda, a national ethics review committee, etc. The formulae used for calculating the indices for the sub-functions are very similar to those used by the United Nations Development Programme to calculate the human development index [20] and the health development governance index [21]. For instance, the national policy on research for health index (R4HPI) could be calculated as follows:

$$ R4HPI=\left(\frac{Actual\ R4HP - Minimum\ R4HP}{Maximum\ R4HP- Minimum\ R4HP}\right) $$

where *Actual R4HP* is the actual research for health policy score, *Minimum R4HP* is the minimum research for health policy score, and *Maximum R4HP* is the maximum research for health policy score. As an example, if we assume that the regional minimum R4HP score is 0 and the maximum score is 100, a country like Uganda would have an actual score of 100, since it has an R4HP. The R4HPI for Uganda would be obtained as follows:

$$ R4HPI=\left(\frac{100 - 0}{100-0}\right)=1. $$

Similarly, the index for progress towards the target of allocating 2% of the national health budget (NHB) to R4H (NHBI) was estimated as follows:

$$ NHBI=\left(\frac{Actual\ NHB - Minimum\ NHB}{Maximum\ NHB- Minimum\ NHB}\right) $$

where *Actual NHB* is the budget allocated for R4H score (i.e. the ratio of the money budgeted by the MOH for research in the last financial year (2012/2013) to the overall MOH budget multiplied by 100%), *Minimum NHB* is the minimum budget allocated for R4H score in the Region, and *Maximum NHB* is the maximum budget allocated for R4H score in the Region. For example, if we assume that the regional minimum NHB score is 0.0104 (Senegal’s score), the maximum NHB score will be 3.2403 (Tanzania’s score), and the actual NHB score for Uganda will be 0.46% (i.e. UGX 1.218 billion for R4H divided by UGX 264.992 billion total budget multiplied by 100). The NHBI for Uganda can be obtained as follows:

$$ NHBI=\left(\frac{0.46 - 0.0104}{3.2403-0.0104}\right) = 0.139. $$

The index for R4H peer-reviewed publications per 100,000 population (PPCI) was estimated as follows:

$$ PPCI=\left(\frac{Actual\ PPC - Minimum\ PPC}{Maximum\ PPC- Minimum\ PPC}\right) $$

where *Actual PPC* is the R4H publications per 100,000 population score (i.e. total R4H publications divided by the country’s population multiplied by 100,000 population), *Minimum PPC* is the minimum number of publications per 100,000 population in the Region, and *Maximum PPC* is the maximum publications per 100,000 population in the Region. For example, if we assume that the regional minimum PPC score to be 0.026225 (Democratic Republic of the Congo’s score) and the maximum PPC score to be 14.9 (Seychelles’ score), and we consider that the actual PPC score for South Africa is 5.9806, the PPCI for South Africa can be obtained as follows:

$$ PPCI=\left(\frac{5.9806 - 0.026225}{14.9-0.026225}\right) = 0.40033. $$

The indices for all the sub-functions were estimated in a similar way.

#### Step 6: Calculate the overall NHRS barometer score for individual countries

After appraising the individual sub-function indices, we calculated the overall NHRS barometer score (*NHRSB*_*Score*_) for each country as follows:

$$ NHRS{B}_{Score}=\left(\frac{{\displaystyle {\sum}_{i=1}^{17}}SFI}{T{N}_{SF}}\right)=\left(\frac{x}{17}\right) = y\ x\ 100\%. $$

where SFI is the sub-function index, ${\displaystyle \sum_{i=1}^{17}}SFI$ is the summation of R4H sub-functions 1 to 17, and *TN*_*SF*_ is the total R4H sub-functions, which is 17 in this study. The national *NHRSB*_*Score*_ is measured on a scale of 0 (or 0%) to 1 (or 100%). A barometer score of 0% denotes that NHRS does not exist, 1% to 49% indicates that NHRS performance is below average, 50% suggests that NHRS performance is average, 51% to 99% shows that NHRS performance is above average, and 100% implies that NHRS is performing optimally.

For instance, the *NHRSB*_*Score*_ for Tanzania was obtained as follows:

$$ \begin{array}{l}NHR{S}_{BScore}=\left(\frac{1+1+1+1+1+1+1+0.001969+1+1+0.00002+0+1+1+1+1+0.054577}{17}\right)\\ {}=\left(\frac{13.06}{17}\right) = 0.77\ x\ 100\%=77\%.\end{array} $$

#### Step 7: Calculate the regional average NHRS sub-function scores

The average regional health research policy index (*RHRP I*_*AVER*_) was calculated using the following formula:

$$ RHRP{I}_{AVER\  Score}=\left(\frac{{\displaystyle {\sum}_{i=1}^{47}} NHRPI}{N}\right) = \left(\frac{x}{47}\right) = y\ x\ 100=Z\% $$

where *RHRPI*_*AVER Score*_ is the average regional health research policy index, NHRPI is the national health research policy index, ${\displaystyle \sum_{i=1}^{47}} NHRPI$ is the summation of NHRPI for countries 1 to 47, and *N* is the total number of Member States in the African Region, which is 47.

$$ RHRP{I}_{AVER\  Score}=\left(\frac{{\displaystyle {\sum}_{i=1}^{47}} NHRPI}{N}\right) = \left(\frac{23}{47}\right) = 0.49\ x\ 100=49\% $$

Since the *RHRPI*_*AVER Score*_ is measured on a scale of 0 (or 0%) to 1 (or 100%), a barometer score of 0.49 (or 49%), for example, indicates that more than half of the Member States did not have a national health research policy. The regional indices for the other sub-functions were calculated in the same manner.

#### Step 8: Calculate the overall regional R4H barometer score

The overall regional health research system barometer score (*RHRSB*_*Score*_) or index is equal to the sum of the average regional indices for the 17 sub-functions divided by 17. This can be expressed symbolically as follows:

$$ RHRS{B}_{Score}=\left(\frac{\begin{array}{c}\hfill RHRPI+ RHRLI+ RHRPI+ RHRMFI+ RERCI+ RHRPLI+\hfill \\ {}\hfill RHRFPI+ RUFHSI+ RHRCI+ RHRPRI+ RHRHRI+\hfill \\ {}\hfill \hfill \\ {}\hfill RHRPAI+ RKTPI+ RBLHRI+ RHRBI+ RNGOI+ RPPCI\hfill \end{array}}{T{N}_{SF}}\right) $$

where: *TN*_*SF*_ is the total health research system sub-functions included in the barometer, which are regional health research policy index (RHRPI), regional health research law index (RHRLI), regional strategic health research plan index (RSHRPI), regional health research management forum index (RHRMFI), regional ethical review committee index (RERCI), regional health research priority list index (RHRPLI), regional health research focal point index (RHRFPI), regional universities with faculties of health sciences/medicine index (RUFHSI), regional health research institutes or council index (RHRCI), regional R4H programme index (RHRPRI), regional R4H programme staff index (RHRHRI), regional R4H programme action plan index (RHRPAI), regional knowledge translation platform index (RKTPI), regional budget line for R4H index (RBLHRI), regional government spending on R4H index (RHRBI), regional non-governmental organisations R4H index (RNGOI), and regional publications per 100,000 population index (RPPCI).

The regional health research system barometer score equation can be summarised as follows:

$$ RHRS{B}_{Score}=\left(\frac{{\displaystyle {\sum}_{i=1}^{17}} RSFI}{T{N}_{SF}}\right)=\left(\frac{x}{17}\right) = y\ x\ 100=K\% $$

where RSFI is the regional sub-function index, ${\displaystyle \sum_{i=1}^{17}} RSFI$ is the summation of the regional R4H sub-functions 1 to 17, and *TN*_*SF*_ is the total R4H sub-functions, which is 17 in this study.

### Ethical clearance

The research proposal and the questionnaire used to collect the NHRS information were approved by the WHO Regional Office for Africa’s ethics review committee. The respondent to the questionnaire in each country was the national health research focal person, to whom the WHO country office research focal person (1) hand-delivered the questionnaire and explained the aims of the survey; (2) gave assurance of the confidentiality of the process and that the survey did not entail any risks; (3) clarified that no benefits would accrue to respondents individually but the survey would inform the development of a NHRS barometer and a regional strategy; and (4) specified the right to refuse to participate in the survey and that refusal to do so would not have a negative impact of any kind. The informed consent statement was included in the preamble to the survey questionnaire, and the respondents were required to sign it.

## Results

### Overall regional NHRS performance

The Region’s R4H systems were categorised as below average if their barometer score was less than 50%, average if the score was 50%, and above average if the score was over 50%. Table 1 presents the regional overall R4H barometer score (*RHRSB*_*Score*_), the average score for each of the research system functions, and the average score for each of the sub-functions. The overall R4H barometer score for the Region was 0.42 (or 42%, ± SD20), which indicates that the performance of NHRSs in the African Region is below average. To understand the source of the poor score, the performance of each NHRS function and its related sub-functions was further examined.

### Governance of R4H

The average regional score for governance of R4H was 61%, which is above average. Within governance, the indices for ethical review committees and the health research focal points were both above 80%, but those for health research policy, research law, and strategic health research plan were all below average.

### Developing and sustaining resources for R4H

The mean regional score for this function was 35%, which is far below average. The indices for health research institutes (or council) and R4H programme were slightly above 50%, but those for universities with faculties of health sciences and R4H programme staff density were below average, with the latter being extremely low at 0.06%. This depicts the acute shortage of human resources for health research in the African Region.

### Producing and using research

The average score for producing and using research was 32%. The index for health research management forum was above average, but those for R4H programme action plan, knowledge translation platform and publications per 100,000 population were less than 35%.

### Financing of R4H

The financing of R4H function had a mean score of 27%. The index for the budget line for R4H was 47% and that for government allocating 2% of national health budget to R4H was only 6%.

### Individual countries’ NHRS performance

Among the 47 countries, the average performance of NHRS was less than 20% for 10 countries, 20–40% for 11, 41–60% for 16, 61–80% for nine, and over 80% for one. The performance of NHRS in 30 (64%) of the countries in the Region was below average. Additional file 1 presents the NHRS sub-functions, functions and overall barometer scores for each of the 47 countries of the African Region.

To assess NHRS performance based on income, the countries were categorised under three income groups: Group 1 consisted of nine high- and upper middle-income countries, group 2 of 13 lower middle-income countries, and group 3 of 25 low-income countries (Table 2). Group 1 had an average NHRSB score of 37% (standard deviation = 24), and its median was 24%. The average score varied greatly, ranging from 13% for Equatorial Guinea, a high-income country, to 79% for South Africa, an upper middle-income country. The average NHRSB score for group 2 was 42% (standard deviation = 17) and the median was 42%. The average score varied significantly, ranging from 6% for Sao Tome and Principe to 71% for Senegal. The average NHRSB score for group 3 was 44% (standard deviation = 21) and the median was 42%. The average score varied remarkably, going from 12% for Chad to 81% for Rwanda. Thus, contrary to expectation, group 3 had a higher average NHRSB score than groups 1 or 2. Groups 2 and 3 had a similar median score of 42%, which was higher than that of group 1.

To determine the relationship between the NHRSB score and the gross domestic product (GDP) per capita, a regression was undertaken of the logarithm of NHRSB scores (logTHEPC) against the logarithm of GDP per capita (logGDPPC) to test the null hypothesis that there was no statistically significant relationship at 95% confidence level. The coefficient of determination (R-squared) was 0.0559, implying that GDP per capita explained only about 6% of the total variation in the NHRSB scores. The results of the log-linear regression were as follows:

$$ \begin{array}{l} log\left( NHRS{B}_{Score}\right) = 2.011955\ \mathit{\hbox{--}}\  0.1330785 log GDPPC.\\ {}\left(\mathrm{t} = 7.27\right)\ \left(\mathrm{t} = -1.63\right)\end{array} $$

Since the computed ‘t’ value (1.63) of *logGDPPC* was less than the critical ‘t’ value of 1.960 (from the Student’s t-distribution table) at 95% confidence level (a significance level of 5%), we accept the null hypothesis that there is no statistically significant relationship between the GDP per capita and the NHRSB score. The coefficient for GDP per capita was −0.133, implying that, on average, a unit percentage increase in the GDP per capita will result in a −0.133 percentage decline in NHRSB. This means that, in African countries, GDP per capita currently does not influence NHRS performance.

## Discussion

This paper has developed an African health systems barometer as a tool for use in identifying gaps in monitoring of NHRS development and performance in the Region. The barometer consists of the four standard functions of an NHRS and 17 sub-functions. The country and regional sub-function NHRS barometer scores would be useful in monitoring the progress in the implementation of the new African Region’s strategy on research for health that was adopted in November 2015 by the ministers of health [17, 22].

As reported by Kirigia et al. [18], between 2009 and March 2014, the African Region experienced a growth of 19% in countries with an official health research policy, 24% in countries with a law regulating R4H, 27% in countries with a national strategic health research plan, 11% in countries reporting a functional NHRS, 7% in countries reporting to have a national health research focal point, and 16% in countries with a national ethics review committee. Despite such improvement in the state of NHRS, various NHRS functions and sub-functions were still weak [23–26]. For instance, in terms of governance of NHRS, 51% of countries did not have an official health research policy, 60% did not have law regulating R4H, 53% did not have a national strategic health research plan, 17% did not have a national health research focal point, 9% did not have a national ethical review committee, and 49% reported they did not have a functional national health research system. Concerning creating and sustaining R4H resources, 13% of countries did not have a university/college of health sciences conducting research, 45% had no national health research institute or council, 49% had no health research programme at the MOH, and 36% had no NGO conducting R4H. Regarding producing and utilizing R4H, 66% of countries with a research programme at the MOH did not have an action plan, 68% did not have a knowledge translation platform, and 49% had no health research management forum. Pertaining to financing for R4H, 53% of the countries had no budget line in the health budget for R4H and 94% of countries had not met the Algiers Ministerial declaration target of allocating at least 2% of the national health budget to R4H.

The overall average NHRS performance in the African Region was 42% (standard deviation = 20) as was the median score, which was below average. The NHRS score varied widely, ranging from 6% for Sao Tome and Principe, a lower middle-income country, to 81% for Rwanda, a low-income country. In the NHRSB score ranking based on 2014 data, the top five countries were Rwanda, Senegal, South Africa, Tanzania and Uganda, each with a score above 70%. The bottom five countries were Equatorial Guinea, Chad, Seychelles, South Sudan, and Sao Tome and Principe, each with a score below 18%.

Our finding that GDP does not significantly or positively influence NHRSB performance is of interest and with implications. One implication is that there is poor allocation of existing funds to R4H, which might be due to factors such as lack of political will, inexistence of R4H governance structures (including policy and legal frameworks), and unawareness of the positive link between R4H and health development with economic prosperity [27]. Thus, although GDP has been shown to be a statistically significant predictor of a country’s volume of research publications [19, 28], it is not necessarily a significant determinant of the overall performance of an NHRS. It is not surprising, therefore, that Africa’s percentage contribution to the global research output based on the publications in international databases is very low (currently only 1.3%) [19].

### Limitations

A few limitations of this study warrant clarification. The NHRSB suffers the same kind of limitations inherent in other types of indices such as the human development index [29, 30]. It does not allow assessment of the quality of sub-functions. The questionnaire upon which the sub-functions barometer scores are based captures only the quantitative aspects of the NHRS as opposed to quality dimensions. For example, the question concerning the national policy on research for health asked ‘Does the country have an official health research policy?’ The questionnaire did not enquire whether the health research policy document – where it existed – had all the policy document elements described in Kirigia et al. [18]. In addition, data from some countries may not be reliable and may be difficult to confirm, though we collected relevant documented evidence when applicable.

The sub-function on R4H publications per 100,000 population took into account only articles indexed in PubMed and other international databases [16]. It excluded books, chapters in books and unpublished literature such as reports of research institutions. This might have introduced a bias in scoring of this sub-function, since the culture of publishing in indexed journals is not uniformly practised among the countries of the Region. Nevertheless, it underpins the fact that publishing in scientifically visible media that are peer-reviewed is the gold standard, as it not only validates the data but also contributes to knowledge, which is especially important in this era of global knowledge sharing and management. Moreover, the fact that publishing in peer-reviewed journals is a monitoring tool to be used periodically in a country or region to assess the sub-functions we used it for in this study, makes it adequate for that purpose.

Finally, the questionnaire used to collect the data on most of the sub-functions had dichotomous (yes or no) questions, which would not capture the qualities of the variables assessed. For example, it is likely that the quality of research policies or any other sub-function of NHRS will vary among the countries. Further, the data on spending on R4H were not completed in the questionnaire for many countries.

Though NHRSB is far from perfect, it provides an indication of the performance of an NHRS in terms of sub-function scores. An objective parameter to identify gaps and monitor progress is always useful, particularly when the same tools are used to compare different time points in the same region or country. It is widely believed that, apart from the enormous investments by countries and international partners, periodic assessment of country performance on the MDGs helped to identify the parameters that needed strengthening and induced positive competition amongst countries. Given the importance of health research, particularly in the African Region, to optimise the use of existing interventions, identify new tools and assess progress towards the newly embarked-on goal of universal health coverage, any element that can enhance R4H would be useful.

In spite of the abovementioned limitations, the NHRSB has a number of advantages: (1) it combines estimates for all the 17 sub-functions of NHRS, spanning the four functions of NHRS and, thus, generating a wide range of information; (2) it uses data that are likely to be readily available in the public domain such as government reports and offices; (3) it allows countries to be ranked in order of their achievements in NHRS development; (4) it allows the comparison of the performance of one sub-function against another; (5) it can be used to help diagnose the sources of weaknesses within an NHRS; (6) it provides a framework for scoring the performance of functions and sub-functions of NHRS; (7) it is largely factual since it does not weigh sub-function scores, which would engender subjectivity; (8) it can be used for cross-country comparisons and benchmarking and for tracking individual countries’ progress over time; and (9) it can highlight the countries with different NHRSB scores but the same per capita GDP, which may imply lurking inefficiency.

The African region strategy on research for health recommends that countries establish national health research and development observatories and registries to facilitate setting of health research priorities [17]. For example, South Africa has a National Health Research Database that allows the National Department of Health and provinces to monitor research activities and use the information to guide the setting of the country’s research agenda [31]. The establishment of research and development observatories, registries and/or databases would, in addition, enable countries to provide the information needed in the development of the African NHRSB to monitor progress in implementation of the regional strategy on R4H.

### Suggestions for future research

We suggest four areas for future research. First, criteria for assessing the quality of specific sub-functions in individual countries should be developed and key informants should use the criteria to score the sub-functions on a scale of 0% to 100%. Second, a survey to determine whether the sub-functions of the NHRS barometer should be weighted should be conducted. Third, R4H accounts should be performed to obtain comprehensive estimates of domestic and external resources spent on NHRS using, for example, the methodology proposed in the Organization of Economic Cooperation and Development Frascati manual [32, 33]. Fourth, test the validity and reliability of the responses to questions on sub-function scores. Reliability is the extent to which participants replicate their initial responses when they are asked to repeat each valuation task. Reliability might be assessed by having a number of persons in each country separately fill out the questionnaire, and convergent validity can be assessed by exploring the covariance between sub-function scores.

## Conclusion

Evidence is urgently needed to guide African countries in their quest for universal health coverage in the context of the unfinished health MDG agenda and the post-2015 Sustainable Development Goals. Generation of appropriate home-grown evidence calls for the existence of effectively performing NHRSs. The weakest NHRS indices in the Region are R4H human resources, government spending on R4H, publications in peer-reviewed journals, and availability of institutions conducting health research. NHRS scores for individual sub-functions can guide policymakers to locate the sources of poor performance so that interventions for their improvement can be designed. The scores will also facilitate the monitoring of NHRS development and performance in the Region.

## Additional file

Additional file 1: National Health Research System sub-function, function and overall barometer scores for each of the 47 countries of the African Region. (DOCX 107 kb)
