# Supplementary material for: Developing the African national health research systems barometer
Source: Health Res Policy Syst. 2016 Jul 22;14:53. doi: 10.1186/s12961-016-0121-4 (PMC4957896; doi:10.1186/s12961-016-0121-4)
Supplement: Additional file 1: — National Health Research System sub-function, function and overall barometer scores for each of the 47 countries of the African Region. (DOCX 107 kb) [file 12961_2016_121_MOESM1_ESM.docx]

| **Additional File 1: National Health Research System sub-function, function and overall barometer scores for each of the 47 countries of the African Region.** | |
| --- | --- |
| **Algeria Health Research System Barometer Scores** |  |
| **Health Research System Barometer Parameters** | **Barometer Score (%)** |
| ***(A). Governance of research for health*** |  |
| (1)   National Health Research Policy Index (HRPI) | 100 |
| (2)   National Health Research Law Index (HRLI) | 100 |
| (3)   National Strategic Health Research Plan Index (SHRPI) | 0 |
| (4)   National Ethical Review Committee (ERCI) Index | 100 |
| (5)   National Health Research Priority List Index (HRPLI) | 100 |
| (6)   National Health Research Focal Point Index (HRFPI) | 100 |
| *Average score for the governance of R4H* | 83.3 |
| ***(B). Developing and sustaining resources for R4H*** |  |
| (7)   National Universities with Faculties of Health Sciences/Medicine (UFHSI) | 1.9 |
| (8)   National Health Research Institutes or Council (HRCI) | 100.0 |
| (9)   National R4H Programme (HRPRI) | 100.0 |
| (10) National R4H Programme Staff Density Index (RHRHRI) | 0.1 |
| (11) National NGOs R4H Index (NGOI) | 0.0 |
| *Average score for developing & sustaining resources for R4H* | 40.4 |
| ***(C). Producing and using research*** |  |
| (12)National R4H Programme Action Plan Index (HRPAI) | 100.0 |
| (13) National Knowledge Translation Platform Index (KTPI) | 0.0 |
| (14) National Health Research Management Forum Index (HRMFI) | 100.0 |
| (15) National R4H Publications Per 100 000 population Index (PAPI)* | 2.8 |
| *Average Score for Producing and Using Research* | 50.7 |
| ***(D). Financing of R4H*** |  |
| (16)National Budget Line for R4H Index (RBLHRI) | 100.0 |
| (17) National Government Spending on R4H Index (RHRBI) | 0.4 |
| *Average Score for Financing of R4H* | 50.2 |
| **National Health Research Systems Average Score** | 59.1 |
|  |  |
| **Angola Health Research System Barometer Scores** |  |
| **Health Research System Barometer Parameters** | **Barometer Score (%)** |
| ***(A). Governance of research for health*** |  |
| (1)   National Health Research Policy Index (HRPI) | 0 |
| (2)   National Health Research Law Index (HRLI) | 0 |
| (3)   National Strategic Health Research Plan Index (SHRPI) | 100 |
| (4)   National Ethical Review Committee (ERCI) Index | 100 |
| (5)   National Health Research Priority List Index (HRPLI) | 0 |
| (6)   National Health Research Focal Point Index (HRFPI) | 100 |
| *Average score for the governance of R4H* | 50 |
| ***(B). Developing and sustaining resources for R4H*** |  |
| (7)   National Universities with Faculties of Health Sciences/Medicine (UFHSI) | 1.584 |
| (8)   National Health Research Institutes or Council (HRCI) | 100 |
| (9)   National R4H Programme (HRPRI) | 100 |
| (10) National R4H Programme Staff Density Index (RHRHRI) | 0.023 |
| (11) National NGOs R4H Index (NGOI) | 100 |
| *Average score for developing & sustaining resources for R4H* | 60.321 |
| ***(C). Producing and using research*** |  |
| (12)National R4H Programme Action Plan Index (HRPAI) | 100 |
| (13) National Knowledge Translation Platform Index (KTPI) | 0 |
| (14) National Health Research Management Forum Index (HRMFI) | 100 |
| (15) National R4H Publications Per 100 000 population Index (PAPI)* | 0.645 |
| *Average Score for Producing and Using Research* | 50.161 |
| ***(D). Financing of R4H*** |  |
| (16)National Budget Line for R4H Index (RBLHRI) | 0 |
| (17) National Government Spending on R4H Index (RHRBI) | 0 |
| *Average Score for Financing of R4H* | 0 |
| **National Health Research Systems Average Score** | 47.2 |
|  |  |
| **Botswana Health Research System Barometer Scores** |  |
| **Health Research System Barometer Parameters** | **Barometer Score (%)** |
| ***(A). Governance of research for health*** |  |
| (1)   National Health Research Policy Index (HRPI) | 100 |
| (2)   National Health Research Law Index (HRLI) | 0 |
| (3)   National Strategic Health Research Plan Index (SHRPI) | 100 |
| (4)   National Ethical Review Committee (ERCI) Index | 100 |
| (5)   National Health Research Priority List Index (HRPLI) | 100 |
| (6)   National Health Research Focal Point Index (HRFPI) | 100 |
| *Average score for the governance of R4H* | 83.33 |
| ***(B). Developing and sustaining resources for R4H*** |  |
| (7)   National Universities with Faculties of Health Sciences/Medicine (UFHSI) | 2.5 |
| (8)   National Health Research Institutes or Council (HRCI) | 0 |
| (9)   National R4H Programme (HRPRI) | 100 |
| (10) National R4H Programme Staff Density Index (RHRHRI) | 0 |
| (11) National NGOs R4H Index (NGOI) | 100 |
| *Average score for developing & sustaining resources for R4H* | 40.5 |
| ***(C). Producing and using research*** |  |
| (12)National R4H Programme Action Plan Index (HRPAI) | 0 |
| (13) National Knowledge Translation Platform Index (KTPI) | 0 |
| (14) National Health Research Management Forum Index (HRMFI) | 100 |
| (15) National R4H Publications Per 100 000 population Index (PAPI)* | 30.25 |
| *Average Score for Producing and Using Research* | 32.56 |
| ***(D). Financing of R4H*** |  |
| (16)National Budget Line for R4H Index (RBLHRI) | 100 |
| (17) National Government Spending on R4H Index (RHRBI) | 0 |
| *Average Score for Financing of R4H* | 50 |
| **National Health Research Systems Average Score** | **54.87** |
|  |  |
| **Burkina Faso Health Research System Barometer Scores** |  |
| **Health Research System Barometer Parameters** | **Barometer Score (%)** |
| ***(A). Governance of research for health*** |  |
| (1)   National Health Research Policy Index (HRPI) | 100 |
| (2)   National Health Research Law Index (HRLI) | 100 |
| (3)   National Strategic Health Research Plan Index (SHRPI) | 100 |
| (4)   National Ethical Review Committee (ERCI) Index | 100 |
| (5)   National Health Research Priority List Index (HRPLI) | 100 |
| (6)   National Health Research Focal Point Index (HRFPI) | 100 |
| *Average score for the governance of R4H* | 100 |
| ***(B). Developing and sustaining resources for R4H*** |  |
| (7)   National Universities with Faculties of Health Sciences/Medicine (UFHSI) | 0.86 |
| (8)   National Health Research Institutes or Council (HRCI) | 100 |
| (9)   National R4H Programme (HRPRI) | 100 |
| (10) National R4H Programme Staff Density Index (RHRHRI) | 0 |
| (11) National NGOs R4H Index (NGOI) | 100 |
| *Average score for developing & sustaining resources for R4H* | 60.17 |
| ***(C). Producing and using research*** |  |
| (12)National R4H Programme Action Plan Index (HRPAI) | 0 |
| (13) National Knowledge Translation Platform Index (KTPI) | 0 |
| (14) National Health Research Management Forum Index (HRMFI) | 100 |
| (15) National R4H Publications Per 100 000 population Index (PAPI)* | 5.99 |
| *Average Score for Producing and Using Research* | 26.50 |
| ***(D). Financing of R4H*** |  |
| (16)National Budget Line for R4H Index (RBLHRI) | 100 |
| (17) National Government Spending on R4H Index (RHRBI) | 1.12 |
| *Average Score for Financing of R4H* | 50.56 |
| **National Health Research Systems Average Score** | **65.18** |
|  |  |
| **Burundi Health Research System Barometer Scores** |  |
| **Health Research System Barometer Parameters** | **Barometer Score (%)** |
| ***(A). Governance of research for health*** |  |
| (1)   National Health Research Policy Index (HRPI) | 0 |
| (2)   National Health Research Law Index (HRLI) | 0 |
| (3)   National Strategic Health Research Plan Index (SHRPI) | 0 |
| (4)   National Ethical Review Committee (ERCI) Index | 100 |
| (5)   National Health Research Priority List Index (HRPLI) | 0 |
| (6)   National Health Research Focal Point Index (HRFPI) | 100 |
| *Average score for the governance of R4H* | 33.333 |
| **(B). Developing and sustaining resources for R4H** |  |
| (7)   National Universities with Faculties of Health Sciences/Medicine (UFHSI) | 0.476 |
| (8)   National Health Research Institutes or Council (HRCI) | 100 |
| (9)   National R4H Programme (HRPRI) | 0 |
| (10) National R4H Programme Staff Density Index (RHRHRI) | 0 |
| (11) National NGOs R4H Index (NGOI) | 100 |
| *Average score for developing & sustaining resources for R4H* | 40.095 |
| **(C). Producing and using research** |  |
| (12)National R4H Programme Action Plan Index (HRPAI) | 0 |
| (13) National Knowledge Translation Platform Index (KTPI) | 0 |
| (14) National Health Research Management Forum Index (HRMFI) | 100 |
| (15) National R4H Publications Per 100 000 population Index (PAPI)* | 0.509 |
| Average Score for Producing and Using Research | 25.127 |
| **(D). Financing of R4H** |  |
| (16)National Budget Line for R4H Index (RBLHRI) | 100 |
| (17) National Government Spending on R4H Index (RHRBI) | 2.169 |
| *Average Score for Financing of R4H* | ***51.084*** |
| **National Health Research Systems Average Score** | **35.48** |
|  |  |
| **Benin Health Research System Barometer Scores** |  |
| **Health Research System Barometer Parameters** | **Barometer Score (%)** |
| ***(A). Governance of research for health*** |  |
| (1)   National Health Research Policy Index (HRPI) | 100 |
| (2)   National Health Research Law Index (HRLI) | 100 |
| (3)   National Strategic Health Research Plan Index (SHRPI) | 100 |
| (4)   National Ethical Review Committee (ERCI) Index | 100 |
| (5)   National Health Research Priority List Index (HRPLI) | 100 |
| (6)   National Health Research Focal Point Index (HRFPI) | 100 |
| *Average score for the governance of R4H* | 100 |
| ***(B). Developing and sustaining resources for R4H*** |  |
| (7)   National Universities with Faculties of Health Sciences/Medicine (UFHSI) | 0.943 |
| (8)   National Health Research Institutes or Council (HRCI) | 0 |
| (9)   National R4H Programme (HRPRI) | 0 |
| (10) National R4H Programme Staff Density Index (RHRHRI) | 0.028 |
| (11) National NGOs R4H Index (NGOI) | 100 |
| *Average score for developing & sustaining resources for R4H* | 20.194 |
| ***(C). Producing and using research*** |  |
| (12)National R4H Programme Action Plan Index (HRPAI) | 0 |
| (13) National Knowledge Translation Platform Index (KTPI) | 0 |
| (14) National Health Research Management Forum Index (HRMFI) | 100 |
| (15) National R4H Publications Per 100 000 population Index (PAPI)* | 5.634 |
| *Average Score for Producing and Using Research* | 26.408 |
| ***(D). Financing of R4H*** |  |
| (16)National Budget Line for R4H Index (RBLHRI) | 100 |
| (17) National Government Spending on R4H Index (RHRBI) | 3.408 |
| *Average Score for Financing of R4H* | *51.704* |
| **National Health Research Systems Average Score** | **53.53** |
|  |  |
| **Chad Health Research System Barometer Scores** |  |
| **Health Research System Barometer Parameters** | **Barometer Score (%)** |
| ***(A). Governance of research for health*** |  |
| (1)   National Health Research Policy Index (HRPI) | 0 |
| (2)   National Health Research Law Index (HRLI) | 0 |
| (3)   National Strategic Health Research Plan Index (SHRPI) | 0 |
| (4)   National Ethical Review Committee (ERCI) Index | 0 |
| (5)   National Health Research Priority List Index (HRPLI) | 0 |
| (6)   National Health Research Focal Point Index (HRFPI) | 0 |
| *Average score for the governance of R4H* | 0 |
| ***(B). Developing and sustaining resources for R4H*** |  |
| (7)   National Universities with Faculties of Health Sciences/Medicine (UFHSI) | 1.136 |
| (8)   National Health Research Institutes or Council (HRCI) | 0 |
| (9)   National R4H Programme (HRPRI) | 0 |
| (10) National R4H Programme Staff Density Index (RHRHRI) | 0 |
| (11) National NGOs R4H Index (NGOI) | 100 |
| *Average score for developing & sustaining resources for R4H* | 20.227 |
| ***(C). Producing and using research*** |  |
| (12)National R4H Programme Action Plan Index (HRPAI) | 0 |
| (13) National Knowledge Translation Platform Index (KTPI) | 0 |
| (14) National Health Research Management Forum Index (HRMFI) | 100 |
| (15) National R4H Publications Per 100 000 population Index (PAPI)* | 2.783 |
| *Average Score for Producing and Using Research* | 25.696 |
| ***(D). Financing of R4H*** |  |
| (16)National Budget Line for R4H Index (RBLHRI) | 0 |
| (17) National Government Spending on R4H Index (RHRBI) | 0 |
| *Average Score for Financing of R4H* | *0* |
| **National Health Research Systems Average Score** | **12.00** |
|  |  |
| **Cameroun Health Research System Barometer Scores** |  |
| **Health Research System Barometer Parameters** | **Barometer Score (%)** |
| ***(A). Governance of research for health*** |  |
| (1)   National Health Research Policy Index (HRPI) | 0 |
| (2)   National Health Research Law Index (HRLI) | 0 |
| (3)   National Strategic Health Research Plan Index (SHRPI) | 100 |
| (4)   National Ethical Review Committee (ERCI) Index | 100 |
| (5)   National Health Research Priority List Index (HRPLI) | 0 |
| (6)   National Health Research Focal Point Index (HRFPI) | 100 |
| *Average score for the governance of R4H* | 50 |
| **(B). Developing and sustaining resources for R4H** |  |
| (7)   National Universities with Faculties of Health Sciences/Medicine (UFHSI) | 1.096 |
| (8)   National Health Research Institutes or Council (HRCI) | 100 |
| (9)   National R4H Programme (HRPRI) | 0 |
| (10) National R4H Programme Staff Density Index (RHRHRI) | 0 |
| (11) National NGOs R4H Index (NGOI) | 100 |
| *Average score for developing & sustaining resources for R4H* | 40.219 |
| **(C). Producing and using research** |  |
| (12)National R4H Programme Action Plan Index (HRPAI) | 0 |
| (13) National Knowledge Translation Platform Index (KTPI) | 0 |
| (14) National Health Research Management Forum Index (HRMFI) | 100 |
| (15) National R4H Publications Per 100 000 population Index (PAPI)* | 8.110 |
| *Average Score for Producing and Using Research* | 27.027 |
| **(D). Financing of R4H** |  |
| (16)National Budget Line for R4H Index (RBLHRI) | 0 |
| (17) National Government Spending on R4H Index (RHRBI) | 0 |
| *Average Score for Financing of R4H* | *0* |
| **National Health Research Systems Average Score** | 35.836 |
|  |  |
| **Cape Verde Health Research System Barometer Scores** |  |
| **Health Research System Barometer Parameters** | **Barometer Score (%)** |
| ***(A). Governance of research for health*** |  |
| (1)   National Health Research Policy Index (HRPI) | 100 |
| (2)   National Health Research Law Index (HRLI) | 100 |
| (3)   National Strategic Health Research Plan Index (SHRPI) | 0 |
| (4)   National Ethical Review Committee (ERCI) Index | 100 |
| (5)   National Health Research Priority List Index (HRPLI) | 0 |
| (6)   National Health Research Focal Point Index (HRFPI) | 100 |
| *Average score for the governance of R4H* | 66.667 |
| ***(B). Developing and sustaining resources for R4H*** |  |
| (7)   National Universities with Faculties of Health Sciences/Medicine (UFHSI) | 40 |
| (8)   National Health Research Institutes or Council (HRCI) | 100 |
| (9)   National R4H Programme (HRPRI) | 0 |
| (10) National R4H Programme Staff Density Index (RHRHRI) | 0 |
| (11) National NGOs R4H Index (NGOI) | 100 |
| *Average score for developing & sustaining resources for R4H* | 48 |
| **(C). Producing and using research** |  |
| (12)National R4H Programme Action Plan Index (HRPAI) | 0 |
| (13) National Knowledge Translation Platform Index (KTPI) | 100 |
| (14) National Health Research Management Forum Index (HRMFI) | 0 |
| (15) National R4H Publications Per 100 000 population Index (PAPI)* | 8.161 |
| *Average Score for Producing and Using Research* | 27.040 |
| **(D). Financing of R4H** |  |
| (16)National Budget Line for R4H Index (RBLHRI) | 100 |
| (17) National Government Spending on R4H Index (RHRBI) | 0 |
| *Average Score for Financing of R4H* | 50 |
| **National Health Research Systems Average Score** | 49.89 |
|  |  |
| **Central African Republic Health Research System Barometer Scores** | |
| **Health Research System Barometer Parameters** | **Barometer Score (%)** |
| ***(A). Governance of research for health*** |  |
| (1)   National Health Research Policy Index (HRPI) | 0 |
| (2)   National Health Research Law Index (HRLI) | 0 |
| (3)   National Strategic Health Research Plan Index (SHRPI) | 100 |
| (4)   National Ethical Review Committee (ERCI) Index | 0 |
| (5)   National Health Research Priority List Index (HRPLI) | 0 |
| (6)   National Health Research Focal Point Index (HRFPI) | 100 |
| *Average score for the governance of R4H* | *33.333* |
| **(B). Developing and sustaining resources for R4H** |  |
| (7)   National Universities with Faculties of Health Sciences/Medicine (UFHSI) | 1.064 |
| (8)   National Health Research Institutes or Council (HRCI) | 100 |
| (9)   National R4H Programme (HRPRI) | 0 |
| (10) National R4H Programme Staff Density Index (RHRHRI) | 0 |
| (11) National NGOs R4H Index (NGOI) | 0 |
| *Average score for developing & sustaining resources for R4H* | *20.213* |
| **(C). Producing and using research** |  |
| (12)National R4H Programme Action Plan Index (HRPAI) | 0 |
| (13) National Knowledge Translation Platform Index (KTPI) | 0 |
| (14) National Health Research Management Forum Index (HRMFI) | 100 |
| (15) National R4H Publications Per 100 000 population Index (PAPI)* | 2.413 |
| *Average Score for Producing and Using Research* | *25.603* |
| ***(D). Financing of R4H*** |  |
| (16)National Budget Line for R4H Index (RBLHRI) | 100 |
| (17) National Government Spending on R4H Index (RHRBI) | 4.599 |
| *Average Score for Financing of R4H* | *52.299* |
| **National Health Research Systems Average Score** | **29.89** |
|  |  |
| **Comoros Health Research System Barometer Scores** |  |
| **Health Research System Barometer Parameters** | **Barometer Score (%)** |
| ***(A). Governance of research for health*** |  |
| (1)   National Health Research Policy Index (HRPI) | 0 |
| (2)   National Health Research Law Index (HRLI) | 0 |
| (3)   National Strategic Health Research Plan Index (SHRPI) | 0 |
| (4)   National Ethical Review Committee (ERCI) Index | 100 |
| (5)   National Health Research Priority List Index (HRPLI) | 0 |
| (6)   National Health Research Focal Point Index (HRFPI) | 100 |
| *Average score for the governance of R4H* | 33.333 |
| **(B). Developing and sustaining resources for R4H** |  |
| (7)   National Universities with Faculties of Health Sciences/Medicine (UFHSI) | 6.25 |
| (8)   National Health Research Institutes or Council (HRCI) | 0 |
| (9)   National R4H Programme (HRPRI) | 100 |
| (10) National R4H Programme Staff Density Index (RHRHRI) | 0.125 |
| (11) National NGOs R4H Index (NGOI) | 0 |
| *Average score for developing & sustaining resources for R4H* | 21.275 |
| **(C). Producing and using research** |  |
| (12)National R4H Programme Action Plan Index (HRPAI) | 0 |
| (13) National Knowledge Translation Platform Index (KTPI) | 0 |
| (14) National Health Research Management Forum Index (HRMFI) | 0 |
| (15) National R4H Publications Per 100 000 population Index (PAPI)* | 4.278 |
| *Average Score for Producing and Using Research* | 1.069 |
| **(D). Financing of R4H** |  |
| (16)National Budget Line for R4H Index (RBLHRI) | 0 |
| (17) National Government Spending on R4H Index (RHRBI) | 0 |
| *Average Score for Financing of R4H* | 0 |
| **National Health Research Systems Average Score** | 18.27 |
|  |  |
| **Congo Health Research System Barometer Scores** |  |
| **Health Research System Barometer Parameters** | **Barometer Score (%)** |
| ***(A). Governance of research for health*** |  |
| (1)   National Health Research Policy Index (HRPI) | 0 |
| (2)   National Health Research Law Index (HRLI) | 0 |
| (3)   National Strategic Health Research Plan Index (SHRPI) | 0 |
| (4)   National Ethical Review Committee (ERCI) Index | 0 |
| (5)   National Health Research Priority List Index (HRPLI) | 0 |
| (6)   National Health Research Focal Point Index (HRFPI) | 100 |
| *Average score for the governance of R4H* | *16.667* |
| **(B). Developing and sustaining resources for R4H** |  |
| (7)   National Universities with Faculties of Health Sciences/Medicine (UFHSI) | 1.087 |
| (8)   National Health Research Institutes or Council (HRCI) | 100 |
| (9)   National R4H Programme (HRPRI) | 0 |
| (10) National R4H Programme Staff Density Index (RHRHRI) | 0 |
| (11) National NGOs R4H Index (NGOI) | 100 |
| *Average score for developing & sustaining resources for R4H* | *40.217* |
| **(C). Producing and using research** |  |
| (12)National R4H Programme Action Plan Index (HRPAI) | 0 |
| (13) National Knowledge Translation Platform Index (KTPI) | 0 |
| (14) National Health Research Management Forum Index (HRMFI) | 100 |
| (15) National R4H Publications Per 100 000 population Index (PAPI)* | 10.946 |
| *Average Score for Producing and Using Research* | *27.737* |
| **(D). Financing of R4H** |  |
| (16)National Budget Line for R4H Index (RBLHRI) | 0 |
| (17) National Government Spending on R4H Index (RHRBI) | 0 |
| *Average Score for Financing of R4H* | *0* |
| **National Health Research Systems Average Score** | **24.24** |
|  |  |
| **Cote D'Ivoire Health Research System Barometer Scores** |  |
| **Health Research System Barometer Parameters** | **Barometer Score (%)** |
| ***(A). Governance of research for health*** |  |
| (1)   National Health Research Policy Index (HRPI) | 100 |
| (2)   National Health Research Law Index (HRLI) | 0 |
| (3)   National Strategic Health Research Plan Index (SHRPI) | 0 |
| (4)   National Ethical Review Committee (ERCI) Index | 100 |
| (5)   National Health Research Priority List Index (HRPLI) | 100 |
| (6)   National Health Research Focal Point Index (HRFPI) | 100 |
| *Average score for the governance of R4H* | 66.667 |
| **(B). Developing and sustaining resources for R4H** |  |
| (7)   National Universities with Faculties of Health Sciences/Medicine (UFHSI) | 0.481 |
| (8)   National Health Research Institutes or Council (HRCI) | 100 |
| (9)   National R4H Programme (HRPRI) | 0 |
| (10) National R4H Programme Staff Density Index (RHRHRI) | 0 |
| (11) National NGOs R4H Index (NGOI) | 100 |
| *Average score for developing & sustaining resources for R4H* | 40.096 |
| **(C). Producing and using research** |  |
| (12)National R4H Programme Action Plan Index (HRPAI) | 0 |
| (13) National Knowledge Translation Platform Index (KTPI) | 0 |
| (14) National Health Research Management Forum Index (HRMFI) | 0 |
| (15) National R4H Publications Per 100 000 population Index (PAPI)* | 4.446 |
| *Average Score for Producing and Using Research* | 1.111 |
| **(D). Financing of R4H** |  |
| (16)National Budget Line for R4H Index (RBLHRI) | 0 |
| (17) National Government Spending on R4H Index (RHRBI) | 0 |
| *Average Score for Financing of R4H* | 0 |
| **National Health Research Systems Average Score** | 35.58 |
|  |  |
| **DRC Health Research System Barometer Scores** |  |
| **Health Research System Barometer Parameters** | **Barometer Score (%)** |
| ***(A). Governance of research for health*** |  |
| (1)   National Health Research Policy Index (HRPI) | 0 |
| (2)   National Health Research Law Index (HRLI) | 0 |
| (3)   National Strategic Health Research Plan Index (SHRPI) | 0 |
| (4)   National Ethical Review Committee (ERCI) Index | 100 |
| (5)   National Health Research Priority List Index (HRPLI) | 100 |
| (6)   National Health Research Focal Point Index (HRFPI) | 100 |
| *Average score for the governance of R4H* | *50* |
| **(B). Developing and sustaining resources for R4H** | 0 |
| (7)   National Universities with Faculties of Health Sciences/Medicine (UFHSI) | 0.072 |
| (8)   National Health Research Institutes or Council (HRCI) | 100 |
| (9)   National R4H Programme (HRPRI) | 0 |
| (10) National R4H Programme Staff Density Index (RHRHRI) | 0 |
| (11) National NGOs R4H Index (NGOI) | 100 |
| *Average score for developing & sustaining resources for R4H* | *40.014* |
| **(C). Producing and using research** | 0 |
| (12)National R4H Programme Action Plan Index (HRPAI) | 0 |
| (13) National Knowledge Translation Platform Index (KTPI) | 0 |
| (14) National Health Research Management Forum Index (HRMFI) | 100 |
| (15) National R4H Publications Per 100 000 population Index (PAPI)* | 0.000 |
| *Average Score for Producing and Using Research* | *25* |
| **(D). Financing of R4H** | 0 |
| (16)National Budget Line for R4H Index (RBLHRI) | 0 |
| (17) National Government Spending on R4H Index (RHRBI) | 0 |
| *Average Score for Financing of R4H* | *0* |
| **National Health Research Systems Average Score** | **35.30** |
|  |  |
| **Eritrea Health Research System Barometer Scores** |  |
| **Health Research System Barometer Parameters** | **Barometer Score (%)** |
| ***(A). Governance of research for health*** |  |
| (1)   National Health Research Policy Index (HRPI) | 100 |
| (2)   National Health Research Law Index (HRLI) | 0 |
| (3)   National Strategic Health Research Plan Index (SHRPI) | 100 |
| (4)   National Ethical Review Committee (ERCI) Index | 100 |
| (5)   National Health Research Priority List Index (HRPLI) | 100 |
| (6)   National Health Research Focal Point Index (HRFPI) | 100 |
| *Average score for the governance of R4H* | *83.333* |
| **(B). Developing and sustaining resources for R4H** | 0 |
| (7)   National Universities with Faculties of Health Sciences/Medicine (UFHSI) | 1.538 |
| (8)   National Health Research Institutes or Council (HRCI) | 0 |
| (9)   National R4H Programme (HRPRI) | 100 |
| (10) National R4H Programme Staff Density Index (RHRHRI) | 0.015 |
| (11) National NGOs R4H Index (NGOI) | 0 |
| *Average score for developing & sustaining resources for R4H* | *20.311* |
| **(C). Producing and using research** | 0 |
| (12)National R4H Programme Action Plan Index (HRPAI) | 100 |
| (13) National Knowledge Translation Platform Index (KTPI) | 0 |
| (14) National Health Research Management Forum Index (HRMFI) | 0 |
| (15) National R4H Publications Per 100 000 population Index (PAPI)* | 4.571 |
| *Average Score for Producing and Using Research* | *26.143* |
| **(D). Financing of R4H** | 0 |
| (16)National Budget Line for R4H Index (RBLHRI) | 0 |
| (17) National Government Spending on R4H Index (RHRBI) | 0 |
| *Average Score for Financing of R4H* | *0* |
| **National Health Research Systems Average Score** | **41.54** |
|  |  |
| **Ethiopia Health Research System Barometer Scores** |  |
| **Health Research System Barometer Parameters** | **Barometer Score (%)** |
| ***(A). Governance of research for health*** |  |
| (1)   National Health Research Policy Index (HRPI) | 100 |
| (2)   National Health Research Law Index (HRLI) | 0 |
| (3)   National Strategic Health Research Plan Index (SHRPI) | 100 |
| (4)   National Ethical Review Committee (ERCI) Index | 100 |
| (5)   National Health Research Priority List Index (HRPLI) | 100 |
| (6)   National Health Research Focal Point Index (HRFPI) | 100 |
| *Average score for the governance of R4H* | 83.333 |
| **(B). Developing and sustaining resources for R4H** | 0 |
| (7)   National Universities with Faculties of Health Sciences/Medicine (UFHSI) | 0.311 |
| (8)   National Health Research Institutes or Council (HRCI) | 100 |
| (9)   National R4H Programme (HRPRI) | 100 |
| (10) National R4H Programme Staff Density Index (RHRHRI) | 0.518 |
| (11) National NGOs R4H Index (NGOI) | 0 |
| *Average score for developing & sustaining resources for R4H* | 40.166 |
| **(C). Producing and using research** | 0 |
| (12)National R4H Programme Action Plan Index (HRPAI) | 100 |
| (13) National Knowledge Translation Platform Index (KTPI) | 100 |
| (14) National Health Research Management Forum Index (HRMFI) | 100 |
| (15) National R4H Publications Per 100 000 population Index (PAPI)* | 2.767 |
| *Average Score for Producing and Using Research* | 75.692 |
| **(D). Financing of R4H** | 0 |
| (16)National Budget Line for R4H Index (RBLHRI) | 100 |
| (17) National Government Spending on R4H Index (RHRBI) | 0 |
| *Average Score for Financing of R4H* | 50 |
| **National Health Research Systems Average Score** | 64.92 |
|  |  |
| **Equatorial Guinea Health Research System Barometer Scores** | |
| **Health Research System Barometer Parameters** | **Barometer Score (%)** |
| ***(A). Governance of research for health*** |  |
| (1)   National Health Research Policy Index (HRPI) | 0 |
| (2)   National Health Research Law Index (HRLI) | 0 |
| (3)   National Strategic Health Research Plan Index (SHRPI) | 0 |
| (4)   National Ethical Review Committee (ERCI) Index | 100 |
| (5)   National Health Research Priority List Index (HRPLI) | 0 |
| (6)   National Health Research Focal Point Index (HRFPI) | 100 |
| ***Average score for the governance of R4H*** | 33.333 |
| **(B). Developing and sustaining resources for R4H** | 0 |
| (7)   National Universities with Faculties of Health Sciences/Medicine (UFHSI) | 6.25 |
| (8)   National Health Research Institutes or Council (HRCI) | 0 |
| (9)   National R4H Programme (HRPRI) | 0 |
| (10) National R4H Programme Staff Density Index (RHRHRI) | 0 |
| (11) National NGOs R4H Index (NGOI) | 0 |
| *Average score for developing & sustaining resources for R4H* | 1.25 |
| **(C). Producing and using research** | 0 |
| (12)National R4H Programme Action Plan Index (HRPAI) | 0 |
| (13) National Knowledge Translation Platform Index (KTPI) | 0 |
| (14) National Health Research Management Forum Index (HRMFI) | 0 |
| (15) National R4H Publications Per 100 000 population Index (PAPI)* | 7.892 |
| *Average Score for Producing and Using Research* | 1.973 |
| **(D). Financing of R4H** | 0 |
| (16)National Budget Line for R4H Index (RBLHRI) | 0 |
| (17) National Government Spending on R4H Index (RHRBI) | 0 |
| *Average Score for Financing of R4H* | *0* |
| **National Health Research Systems Average Score** | **12.60** |
|  |  |
| **Gabon Health Research System Barometer Scores** |  |
| **Health Research System Barometer Parameters** | **Barometer Score (%)** |
| ***(A). Governance of research for health*** |  |
| (1)   National Health Research Policy Index (HRPI) | 0 |
| (2)   National Health Research Law Index (HRLI) | 0 |
| (3)   National Strategic Health Research Plan Index (SHRPI) | 0 |
| (4)   National Ethical Review Committee (ERCI) Index | 100 |
| (5)   National Health Research Priority List Index (HRPLI) | 0 |
| (6)   National Health Research Focal Point Index (HRFPI) | 0 |
| *Average score for the governance of R4H* | 16.667 |
| **(B). Developing and sustaining resources for R4H** | 0 |
| (7)   National Universities with Faculties of Health Sciences/Medicine (UFHSI) | 2.941 |
| (8)   National Health Research Institutes or Council (HRCI) | 100 |
| (9)   National R4H Programme (HRPRI) | 0 |
| (10) National R4H Programme Staff Density Index (RHRHRI) | 0 |
| (11) National NGOs R4H Index (NGOI) | 0 |
| *Average score for developing & sustaining resources for R4H* | 20.588 |
| **(C). Producing and using research** | 0 |
| (12)National R4H Programme Action Plan Index (HRPAI) | 0 |
| (13) National Knowledge Translation Platform Index (KTPI) | 0 |
| (14) National Health Research Management Forum Index (HRMFI) | 0 |
| (15) National R4H Publications Per 100 000 population Index (PAPI)* | 27.191 |
| *Average Score for Producing and Using Research* | 6.798 |
| **(D). Financing of R4H** | 0 |
| (16)National Budget Line for R4H Index (RBLHRI) | 100 |
| (17) National Government Spending on R4H Index (RHRBI) | 0.990 |
| *Average Score for Financing of R4H* | 50.495 |
| **National Health Research Systems Average Score** | 19.48 |
|  |  |
| **Ghana Health Research System Barometer Scores** |  |
| **Health Research System Barometer Parameters** | **Barometer Score (%)** |
| **(A). Governance of research for health** |  |
| (1)   National Health Research Policy Index (HRPI) | 0 |
| (2)   National Health Research Law Index (HRLI) | 0 |
| (3)   National Strategic Health Research Plan Index (SHRPI) | 100 |
| (4)   National Ethical Review Committee (ERCI) Index | 100 |
| (5)   National Health Research Priority List Index (HRPLI) | 100 |
| (6)   National Health Research Focal Point Index (HRFPI) | 0 |
| *Average score for the governance of R4H* | *50* |
| **(B). Developing and sustaining resources for R4H** | 0 |
| (7)   National Universities with Faculties of Health Sciences/Medicine (UFHSI) | 0.947 |
| (8)   National Health Research Institutes or Council (HRCI) | 0 |
| (9)   National R4H Programme (HRPRI) | 100 |
| (10) National R4H Programme Staff Density Index (RHRHRI) | 0.227 |
| (11) National NGOs R4H Index (NGOI) | 100 |
| *Average score for developing & sustaining resources for R4H* | *40.235* |
| **(C). Producing and using research** | 0 |
| (12)National R4H Programme Action Plan Index (HRPAI) | 100 |
| (13) National Knowledge Translation Platform Index (KTPI) | 100 |
| (14) National Health Research Management Forum Index (HRMFI) | 0 |
| (15) National R4H Publications Per 100 000 population Index (PAPI)* | 8.080 |
| *Average Score for Producing and Using Research* | *52.020* |
| **(D). Financing of R4H** | 0 |
| (16)National Budget Line for R4H Index (RBLHRI) | 100 |
| (17) National Government Spending on R4H Index (RHRBI) | 0.429 |
| *Average Score for Financing of R4H* | *50.214* |
| **National Health Research Systems Average Score** | **47.63** |
|  |  |
| **Gambia Health Research System Barometer Scores** |  |
| **Health Research System Barometer Parameters** | **Barometer Score (%)** |
| ***(A). Governance of research for health*** |  |
| (1)   National Health Research Policy Index (HRPI) | 100 |
| (2)   National Health Research Law Index (HRLI) | 0 |
| (3)   National Strategic Health Research Plan Index (SHRPI) | 100 |
| (4)   National Ethical Review Committee (ERCI) Index | 100 |
| (5)   National Health Research Priority List Index (HRPLI) | 0 |
| (6)   National Health Research Focal Point Index (HRFPI) | 100 |
| *Average score for the governance of R4H* | *66.667* |
| **(B). Developing and sustaining resources for R4H** | 0 |
| (7)   National Universities with Faculties of Health Sciences/Medicine (UFHSI) | 2.632 |
| (8)   National Health Research Institutes or Council (HRCI) | 0 |
| (9)   National R4H Programme (HRPRI) | 100 |
| (10) National R4H Programme Staff Density Index (RHRHRI) | 0.158 |
| (11) National NGOs R4H Index (NGOI) | 100 |
| *Average score for developing & sustaining resources for R4H* | *40.558* |
| **(C). Producing and using research** | 0 |
| (12)National R4H Programme Action Plan Index (HRPAI) | 0 |
| (13) National Knowledge Translation Platform Index (KTPI) | 100 |
| (14) National Health Research Management Forum Index (HRMFI) | 0 |
| (15) National R4H Publications Per 100 000 population Index (PAPI)* | 30.114 |
| *Average Score for Producing and Using Research* | *32.528* |
| **(D). Financing of R4H** | 0 |
| (16)National Budget Line for R4H Index (RBLHRI) | 0 |
| (17) National Government Spending on R4H Index (RHRBI) | 0 |
| *Average Score for Financing of R4H* | *0* |
| **National Health Research Systems Average Score** | **43.11** |
|  |  |
| **Guinea Health Research System Barometer Scores** |  |
| **Health Research System Barometer Parameters** | **Barometer Score (%)** |
| ***(A). Governance of research for health*** |  |
| (1)   National Health Research Policy Index (HRPI) | 100 |
| (2)   National Health Research Law Index (HRLI) | 100 |
| (3)   National Strategic Health Research Plan Index (SHRPI) | 100 |
| (4)   National Ethical Review Committee (ERCI) Index | 100 |
| (5)   National Health Research Priority List Index (HRPLI) | 100 |
| (6)   National Health Research Focal Point Index (HRFPI) | 100 |
| *Average score for the governance of R4H* | *100* |
| ***(B). Developing and sustaining resources for R4H*** | 0 |
| (7)   National Universities with Faculties of Health Sciences/Medicine (UFHSI) | 2.083 |
| (8)   National Health Research Institutes or Council (HRCI) | 100 |
| (9)   National R4H Programme (HRPRI) | 0 |
| (10) National R4H Programme Staff Density Index (RHRHRI) | 0.0167 |
| (11) National NGOs R4H Index (NGOI) | 100 |
| *Average score for developing & sustaining resources for R4H* | *40.42* |
| ***(C). Producing and using research*** | 0 |
| (12)National R4H Programme Action Plan Index (HRPAI) | 0 |
| (13) National Knowledge Translation Platform Index (KTPI) | 0 |
| (14) National Health Research Management Forum Index (HRMFI) | 100 |
| (15) National R4H Publications Per 100 000 population Index (PAPI)* | 2.760 |
| *Average Score for Producing and Using Research* | *25.690* |
| ***(D). Financing of R4H*** | 0 |
| (16)National Budget Line for R4H Index (RBLHRI) | 0 |
| (17) National Government Spending on R4H Index (RHRBI) | 0 |
| *Average Score for Financing of R4H* | *0* |
| **National Health Research Systems Average Score** | 53.23 |
|  |  |
| **Guinea-Bissau Health Research System Barometer Scores** |  |
| **Health Research System Barometer Parameters** | **Barometer Score (%)** |
| ***(A). Governance of research for health*** |  |
| (1)   National Health Research Policy Index (HRPI) | 0 |
| (2)   National Health Research Law Index (HRLI) | 0 |
| (3)   National Strategic Health Research Plan Index (SHRPI) | 0 |
| (4)   National Ethical Review Committee (ERCI) Index | 100 |
| (5)   National Health Research Priority List Index (HRPLI) | 100 |
| (6)   National Health Research Focal Point Index (HRFPI) | 0 |
| *Average score for the governance of R4H* | 33.333 |
| **(B). Developing and sustaining resources for R4H** | 0 |
| (7)   National Universities with Faculties of Health Sciences/Medicine (UFHSI) | 2.941 |
| (8)   National Health Research Institutes or Council (HRCI) | 100 |
| (9)   National R4H Programme (HRPRI) | 0 |
| (10) National R4H Programme Staff Density Index (RHRHRI) | 0 |
| (11) National NGOs R4H Index (NGOI) | 100 |
| *Average score for developing & sustaining resources for R4H* | 40.588 |
| **(C). Producing and using research** | 0 |
| (12)National R4H Programme Action Plan Index (HRPAI) | 0 |
| (13) National Knowledge Translation Platform Index (KTPI) | 0 |
| (14) National Health Research Management Forum Index (HRMFI) | 100 |
| (15) National R4H Publications Per 100 000 population Index (PAPI)* | 12.954 |
| *Average Score for Producing and Using Research* | 28.238 |
| **(D). Financing of R4H** | 0 |
| (16)National Budget Line for R4H Index (RBLHRI) | 0 |
| (17) National Government Spending on R4H Index (RHRBI) | 0 |
| *Average Score for Financing of R4H* | 0 |
| **National Health Research Systems Average Score** | **30.35** |
|  |  |
| **Kenya Health Research System Barometer Scores** |  |
| **Health Research System Barometer Parameters** | **Barometer Score (%)** |
| ***(A). Governance of research for health*** |  |
| (1)   National Health Research Policy Index (HRPI) | 0 |
| (2)   National Health Research Law Index (HRLI) | 100 |
| (3)   National Strategic Health Research Plan Index (SHRPI) | 0 |
| (4)   National Ethical Review Committee (ERCI) Index | 100 |
| (5)   National Health Research Priority List Index (HRPLI) | 0 |
| (6)   National Health Research Focal Point Index (HRFPI) | 100 |
| *Average score for the governance of R4H* | 50 |
| ***(B). Developing and sustaining resources for R4H*** | 0 |
| (7)   National Universities with Faculties of Health Sciences/Medicine (UFHSI) | 0.769 |
| (8)   National Health Research Institutes or Council (HRCI) | 100 |
| (9)   National R4H Programme (HRPRI) | 100 |
| (10) National R4H Programme Staff Density Index (RHRHRI) | 0.011 |
| (11) National NGOs R4H Index (NGOI) | 100 |
| *Average score for developing & sustaining resources for R4H* | *60.156* |
| **(C). Producing and using research** | 0 |
| (12)National R4H Programme Action Plan Index (HRPAI) | 100 |
| (13) National Knowledge Translation Platform Index (KTPI) | 0 |
| (14) National Health Research Management Forum Index (HRMFI) | 0 |
| (15) National R4H Publications Per 100 000 population Index (PAPI)* | 9.6662161 |
| *Average Score for Producing and Using Research* | *27.417* |
| **(D). Financing of R4H** | 0 |
| (16)National Budget Line for R4H Index (RBLHRI) | 0 |
| (17) National Government Spending on R4H Index (RHRBI) | 0.383 |
| *Average Score for Financing of R4H* | *0.191* |
| **National Health Research Systems Average Score** | **41.81** |
|  |  |
| **Lesotho Health Research System Barometer Scores** |  |
| **Health Research System Barometer Parameters** | **Barometer Score (%)** |
| ***(A). Governance of research for health*** |  |
| (1)   National Health Research Policy Index (HRPI) | 100 |
| (2)   National Health Research Law Index (HRLI) | 100 |
| (3)   National Strategic Health Research Plan Index (SHRPI) | 100 |
| (4)   National Ethical Review Committee (ERCI) Index | 100 |
| (5)   National Health Research Priority List Index (HRPLI) | 100 |
| (6)   National Health Research Focal Point Index (HRFPI) | 100 |
| *Average score for the governance of R4H* | 100 |
| **(B). Developing and sustaining resources for R4H** |  |
| (7)   National Universities with Faculties of Health Sciences/Medicine (UFHSI) | 2.381 |
| (8)   National Health Research Institutes or Council (HRCI) | 0 |
| (9)   National R4H Programme (HRPRI) | 0 |
| (10) National R4H Programme Staff Density Index (RHRHRI) | 0.095 |
| (11) National NGOs R4H Index (NGOI) | 100 |
| *Average score for developing & sustaining resources for R4H* | 20.495 |
| **(C). Producing and using research** | 0 |
| (12)National R4H Programme Action Plan Index (HRPAI) | 0 |
| (13) National Knowledge Translation Platform Index (KTPI) | 100 |
| (14) National Health Research Management Forum Index (HRMFI) | 0 |
| (15) National R4H Publications Per 100 000 population Index (PAPI)* | 4.114 |
| *Average Score for Producing and Using Research* | 26.028 |
| **(D). Financing of R4H** |  |
| (16)National Budget Line for R4H Index (RBLHRI) | 0 |
| (17) National Government Spending on R4H Index (RHRBI) | 0 |
| *Average Score for Financing of R4H* | 0 |
| **National Health Research Systems Average Score** | 47.45 |
|  |  |
| **Liberia Health Research System Barometer Scores** |  |
| **Health Research System Barometer Parameters** |  |
| ***(A). Governance of research for health*** |  |
| (1)   National Health Research Policy Index (HRPI) | 0 |
| (2)   National Health Research Law Index (HRLI) | 0 |
| (3)   National Strategic Health Research Plan Index (SHRPI) | 0 |
| (4)   National Ethical Review Committee (ERCI) Index | 100 |
| (5)   National Health Research Priority List Index (HRPLI) | 100 |
| (6)   National Health Research Focal Point Index (HRFPI) | 100 |
| *Average score for the governance of R4H* | *50* |
| **(B). Developing and sustaining resources for R4H** | 0 |
| (7)   National Universities with Faculties of Health Sciences/Medicine (UFHSI) | 3.409 |
| (8)   National Health Research Institutes or Council (HRCI) | 0 |
| (9)   National R4H Programme (HRPRI) | 100 |
| (10) National R4H Programme Staff Density Index (RHRHRI) | 0.114 |
| (11) National NGOs R4H Index (NGOI) | 100 |
| *Average score for developing & sustaining resources for R4H* | *40.705* |
| **(C). Producing and using research** | 0 |
| (12)National R4H Programme Action Plan Index (HRPAI) | 100 |
| (13) National Knowledge Translation Platform Index (KTPI) | 0 |
| (14) National Health Research Management Forum Index (HRMFI) | 0 |
| (15) National R4H Publications Per 100 000 population Index (PAPI)* | 1.718 |
| *Average Score for Producing and Using Research* | *25.430* |
| **(D). Financing of R4H** | 0 |
| (16)National Budget Line for R4H Index (RBLHRI) | 0 |
| (17) National Government Spending on R4H Index (RHRBI) | 0 |
| *Average Score for Financing of R4H* | *0* |
| **National Health Research Systems Average Score** | **35.60** |
|  |  |
| **Madagascar Health Research System Barometer Scores** |  |
| **Health Research System Barometer Parameters** | **Barometer Score (%)** |
| ***(A). Governance of research for health*** |  |
| (1)   National Health Research Policy Index (HRPI) | 100 |
| (2)   National Health Research Law Index (HRLI) | 0 |
| (3)   National Strategic Health Research Plan Index (SHRPI) | 0 |
| (4)   National Ethical Review Committee (ERCI) Index | 100 |
| (5)   National Health Research Priority List Index (HRPLI) | 0 |
| (6)   National Health Research Focal Point Index (HRFPI) | 100 |
| *Average score for the governance of R4H* | 50 |
| **(B). Developing and sustaining resources for R4H** | 0 |
| (7)   National Universities with Faculties of Health Sciences/Medicine (UFHSI) | 1.271 |
| (8)   National Health Research Institutes or Council (HRCI) | 100 |
| (9)   National R4H Programme (HRPRI) | 100 |
| (10) National R4H Programme Staff Density Index (RHRHRI) | 0 |
| (11) National NGOs R4H Index (NGOI) | 0 |
| *Average score for developing & sustaining resources for R4H* | 40.254 |
| **(C). Producing and using research** | 0 |
| (12)National R4H Programme Action Plan Index (HRPAI) | 100 |
| (13) National Knowledge Translation Platform Index (KTPI) | 0 |
| (14) National Health Research Management Forum Index (HRMFI) | 0 |
| (15) National R4H Publications Per 100 000 population Index (PAPI)* | 3.288 |
| *Average Score for Producing and Using Research* | 25.822 |
| **(D). Financing of R4H** | 0 |
| (16)National Budget Line for R4H Index (RBLHRI) | 100 |
| (17) National Government Spending on R4H Index (RHRBI) | 3.044 |
| *Average Score for Financing of R4H* | 51.522 |
| **National Health Research Systems Average Score** | 41.62 |
|  |  |
| **Malawi Health Research System Barometer Scores** |  |
| **Health Research System Barometer Parameters** | **Barometer Score (%)** |
| **(A). Governance of research for health** |  |
| (1)   National Health Research Policy Index (HRPI) | 0 |
| (2)   National Health Research Law Index (HRLI) | 100 |
| (3)   National Strategic Health Research Plan Index (SHRPI) | 0 |
| (4)   National Ethical Review Committee (ERCI) Index | 100 |
| (5)   National Health Research Priority List Index (HRPLI) | 100 |
| (6)   National Health Research Focal Point Index (HRFPI) | 100 |
| *Average score for the governance of R4H* | *66.667* |
| **(B). Developing and sustaining resources for R4H** | 0 |
| (7)   National Universities with Faculties of Health Sciences/Medicine (UFHSI) | 0.298 |
| (8)   National Health Research Institutes or Council (HRCI) | 0 |
| (9)   National R4H Programme (HRPRI) | 100 |
| (10) National R4H Programme Staff Density Index (RHRHRI) | 0.0298 |
| (11) National NGOs R4H Index (NGOI) | 0 |
| *Average score for developing & sustaining resources for R4H* | *20.065* |
| **(C). Producing and using research** | 0 |
| (12)National R4H Programme Action Plan Index (HRPAI) | 100 |
| (13) National Knowledge Translation Platform Index (KTPI) | 100 |
| (14) National Health Research Management Forum Index (HRMFI) | 0 |
| (15) National R4H Publications Per 100 000 population Index (PAPI)* | 9.432 |
| *Average Score for Producing and Using Research* | *52.358* |
| **(D). Financing of R4H** | 0 |
| (16)National Budget Line for R4H Index (RBLHRI) | 100 |
| (17) National Government Spending on R4H Index (RHRBI) | 2.369 |
| *Average Score for Financing of R4H* | *51.185* |
| **National Health Research Systems Average Score** | 47.77 |
|  |  |
| **Mali Health Research System Barometer Scores** |  |
| **Health Research System Barometer Parameters** | **Barometer Score (%)** |
| **(A). Governance of research for health** |  |
| (1)   National Health Research Policy Index (HRPI) | 100 |
| (2)   National Health Research Law Index (HRLI) | 0 |
| (3)   National Strategic Health Research Plan Index (SHRPI) | 100 |
| (4)   National Ethical Review Committee (ERCI) Index | 100 |
| (5)   National Health Research Priority List Index (HRPLI) | 100 |
| (6)   National Health Research Focal Point Index (HRFPI) | 100 |
| *Average score for the governance of R4H* | *83.333* |
| **(B). Developing and sustaining resources for R4H** | 0 |
| (7)   National Universities with Faculties of Health Sciences/Medicine (UFHSI) | 0.316 |
| (8)   National Health Research Institutes or Council (HRCI) | 100 |
| (9)   National R4H Programme (HRPRI) | 100 |
| (10) National R4H Programme Staff Density Index (RHRHRI) | 0.006 |
| (11) National NGOs R4H Index (NGOI) | 100 |
| *Average score for developing & sustaining resources for R4H* | *60.065* |
| **(C). Producing and using research** | 0 |
| (12)National R4H Programme Action Plan Index (HRPAI) | 0 |
| (13) National Knowledge Translation Platform Index (KTPI) | 0 |
| (14) National Health Research Management Forum Index (HRMFI) | 100 |
| (15) National R4H Publications Per 100 000 population Index (PAPI)* | 4.215 |
| *Average Score for Producing and Using Research* | *26.054* |
| **(D). Financing of R4H** | 0 |
| (16)National Budget Line for R4H Index (RBLHRI) | 100 |
| (17) National Government Spending on R4H Index (RHRBI) | 0 |
| *Average Score for Financing of R4H* | *50* |
| **National Health Research Systems Average Score** | **59.09** |
|  |  |
| **Mauritania Health Research System Barometer Scores** |  |
| **Health Research System Barometer Parameters** | **Barometer Score (%)** |
| ***(A). Governance of research for health*** |  |
| (1)   National Health Research Policy Index (HRPI) | 0 |
| (2)   National Health Research Law Index (HRLI) | 0 |
| (3)   National Strategic Health Research Plan Index (SHRPI) | 0 |
| (4)   National Ethical Review Committee (ERCI) Index | 100 |
| (5)   National Health Research Priority List Index (HRPLI) | 0 |
| (6)   National Health Research Focal Point Index (HRFPI) | 100 |
| *Average score for the governance of R4H* | 33.333 |
| **(B). Developing and sustaining resources for R4H** | 0 |
| (7)   National Universities with Faculties of Health Sciences/Medicine (UFHSI) | 3.75 |
| (8)   National Health Research Institutes or Council (HRCI) | 100 |
| (9)   National R4H Programme (HRPRI) | 0 |
| (10) National R4H Programme Staff Density Index (RHRHRI) | 0 |
| (11) National NGOs R4H Index (NGOI) | 0 |
| *Average score for developing & sustaining resources for R4H* | *20.750* |
| **(C). Producing and using research** | 0 |
| (12)National R4H Programme Action Plan Index (HRPAI) | 0 |
| (13) National Knowledge Translation Platform Index (KTPI) | 0 |
| (14) National Health Research Management Forum Index (HRMFI) | 100 |
| (15) National R4H Publications Per 100 000 population Index (PAPI)* | 1.320 |
| *Average Score for Producing and Using Research* | *25.330* |
| **(D). Financing of R4H** | 0 |
| (16)National Budget Line for R4H Index (RBLHRI) | 100 |
| (17) National Government Spending on R4H Index (RHRBI) | 0 |
| *Average Score for Financing of R4H* | *50* |
| **National Health Research Systems Average Score** | 29.71 |
|  |  |
| ***Mauritius Health Research System Barometer Scores*** |  |
| ***Health Research System Barometer Parameters*** |  |
| **(A). Governance of research for health** |  |
| (1)   National Health Research Policy Index (HRPI) | 0 |
| (2)   National Health Research Law Index (HRLI) | 100 |
| (3)   National Strategic Health Research Plan Index (SHRPI) | 0 |
| (4)   National Ethical Review Committee (ERCI) Index | 100 |
| (5)   National Health Research Priority List Index (HRPLI) | 0 |
| (6)   National Health Research Focal Point Index (HRFPI) | 0 |
| *Average score for the governance of R4H* | *33.333* |
| **(B). Developing and sustaining resources for R4H** | 0 |
| (7)   National Universities with Faculties of Health Sciences/Medicine (UFHSI) | 4.167 |
| (8)   National Health Research Institutes or Council (HRCI) | 100 |
| (9)   National R4H Programme (HRPRI) | 0 |
| (10) National R4H Programme Staff Density Index (RHRHRI) | 0 |
| (11) National NGOs R4H Index (NGOI) | 0 |
| *Average score for developing & sustaining resources for R4H* | *20.833* |
| **(C). Producing and using research** | 0 |
| (12)National R4H Programme Action Plan Index (HRPAI) | 0 |
| (13) National Knowledge Translation Platform Index (KTPI) | 0 |
| (14) National Health Research Management Forum Index (HRMFI) | 0 |
| (15) National R4H Publications Per 100 000 population Index (PAPI)* | 16.856 |
| *Average Score for Producing and Using Research* | *4.214* |
| **(D). Financing of R4H** | 0 |
| (16)National Budget Line for R4H Index (RBLHRI) | 0 |
| (17) National Government Spending on R4H Index (RHRBI) | 0 |
| *Average Score for Financing of R4H* | *0* |
| **National Health Research Systems Average Score** | **18.88** |
|  |  |
| **Mozambique Health Research System Barometer Scores** |  |
| **Health Research System Barometer Parameters** | **Barometer Score (%)** |
| ***(A). Governance of research for health*** |  |
| (1)   National Health Research Policy Index (HRPI) | 100 |
| (2)   National Health Research Law Index (HRLI) | 0 |
| (3)   National Strategic Health Research Plan Index (SHRPI) | 0 |
| (4)   National Ethical Review Committee (ERCI) Index | 100 |
| (5)   National Health Research Priority List Index (HRPLI) | 100 |
| (6)   National Health Research Focal Point Index (HRFPI) | 0 |
| *Average score for the governance of R4H* | *50* |
| **(B). Developing and sustaining resources for R4H** | 0 |
| (7)   National Universities with Faculties of Health Sciences/Medicine (UFHSI) | 1.509 |
| (8)   National Health Research Institutes or Council (HRCI) | 100 |
| (9)   National R4H Programme (HRPRI) | 0 |
| (10) National R4H Programme Staff Density Index (RHRHRI) | 0 |
| (11) National NGOs R4H Index (NGOI) | 100 |
| *Average score for developing & sustaining resources for R4H* | *40.302* |
| **(C). Producing and using research** | 0 |
| (12)National R4H Programme Action Plan Index (HRPAI) | 0 |
| (13) National Knowledge Translation Platform Index (KTPI) | 0 |
| (14) National Health Research Management Forum Index (HRMFI) | 0 |
| (15) National R4H Publications Per 100 000 population Index (PAPI)* | 2.330 |
| *Average Score for Producing and Using Research* | *0.583* |
| **(D). Financing of R4H** | 0 |
| (16)National Budget Line for R4H Index (RBLHRI) | 0 |
| (17) National Government Spending on R4H Index (RHRBI) | 0 |
| *Average Score for Financing of R4H* | *0* |
| **National Health Research Systems Average Score** | **29.64** |
|  |  |
| **Namibia Health Research System Barometer Scores** |  |
| **Health Research System Barometer Parameters** | **Barometer Score (%)** |
| ***(A). Governance of research for health*** |  |
| (1)   National Health Research Policy Index (HRPI) | 100 |
| (2)   National Health Research Law Index (HRLI) | 100 |
| (3)   National Strategic Health Research Plan Index (SHRPI) | 0 |
| (4)   National Ethical Review Committee (ERCI) Index | 100 |
| (5)   National Health Research Priority List Index (HRPLI) | 0 |
| (6)   National Health Research Focal Point Index (HRFPI) | 100 |
| *Average score for the governance of R4H* | *66.667* |
| **(B). Developing and sustaining resources for R4H** | 0 |
| (7)   National Universities with Faculties of Health Sciences/Medicine (UFHSI) | 6.522 |
| (8)   National Health Research Institutes or Council (HRCI) | 0 |
| (9)   National R4H Programme (HRPRI) | 0 |
| (10) National R4H Programme Staff Density Index (RHRHRI) | 0.261 |
| (11) National NGOs R4H Index (NGOI) | 0 |
| *Average score for developing & sustaining resources for R4H* | *1.357* |
| **(C). Producing and using research** | 0 |
| (12)National R4H Programme Action Plan Index (HRPAI) | 0 |
| (13) National Knowledge Translation Platform Index (KTPI) | 0 |
| (14) National Health Research Management Forum Index (HRMFI) | 0 |
| (15) National R4H Publications Per 100 000 population Index (PAPI)* | 8.944 |
| *Average Score for Producing and Using Research* | *2.236* |
| **(D). Financing of R4H** | 0 |
| (16)National Budget Line for R4H Index (RBLHRI) | 0 |
| (17) National Government Spending on R4H Index (RHRBI) | 0 |
| *Average Score for Financing of R4H* | *0* |
| **National Health Research Systems Average Score** | **24.45** |
|  |  |
| **Niger Health Research System Barometer Scores** |  |
| **Health Research System Barometer Parameters** | **Barometer Score (%)** |
| ***(A). Governance of research for health*** |  |
| (1)   National Health Research Policy Index (HRPI) | 100 |
| (2)   National Health Research Law Index (HRLI) | 100 |
| (3)   National Strategic Health Research Plan Index (SHRPI) | 100 |
| (4)   National Ethical Review Committee (ERCI) Index | 100 |
| (5)   National Health Research Priority List Index (HRPLI) | 100 |
| (6)   National Health Research Focal Point Index (HRFPI) | 100 |
| *Average score for the governance of R4H* | 100 |
| **(B). Developing and sustaining resources for R4H** |  |
| (7)   National Universities with Faculties of Health Sciences/Medicine (UFHSI) | 0.541 |
| (8)   National Health Research Institutes or Council (HRCI) | 100 |
| (9)   National R4H Programme (HRPRI) | 0 |
| (10) National R4H Programme Staff Density Index (RHRHRI) | 0 |
| (11) National NGOs R4H Index (NGOI) | 100 |
| *Average score for developing & sustaining resources for R4H* | *40.108* |
| **(C). Producing and using research** |  |
| (12)National R4H Programme Action Plan Index (HRPAI) | 0 |
| (13) National Knowledge Translation Platform Index (KTPI) | 100 |
| (14) National Health Research Management Forum Index (HRMFI) | 100 |
| (15) National R4H Publications Per 100 000 population Index (PAPI)* | 6.278 |
| *Average Score for Producing and Using Research* | *51.569* |
| **(D). Financing of R4H** |  |
| (16)National Budget Line for R4H Index (RBLHRI) | 100 |
| (17) National Government Spending on R4H Index (RHRBI) | 0.547 |
| *Average Score for Financing of R4H* | *50.273* |
| **National Health Research Systems Average Score** | **65.14** |
|  |  |
| **Nigeria Health Research System Barometer Scores** |  |
| **Health Research System Barometer Parameters** | **Barometer Score (%)** |
| ***(A). Governance of research for health*** |  |
| (1)   National Health Research Policy Index (HRPI) | 0 |
| (2)   National Health Research Law Index (HRLI) | 0 |
| (3)   National Strategic Health Research Plan Index (SHRPI) | 100 |
| (4)   National Ethical Review Committee (ERCI) Index | 100 |
| (5)   National Health Research Priority List Index (HRPLI) | 100 |
| (6)   National Health Research Focal Point Index (HRFPI) | 100 |
| *Average score for the governance of R4H* | *66.667* |
| **(B). Developing and sustaining resources for R4H** | 0 |
| (7)   National Universities with Faculties of Health Sciences/Medicine (UFHSI) | 0.448 |
| (8)   National Health Research Institutes or Council (HRCI) | 100 |
| (9)   National R4H Programme (HRPRI) | 100 |
| (10) National R4H Programme Staff Density Index (RHRHRI) | 0.005 |
| (11) National NGOs R4H Index (NGOI) | 100 |
| *Average score for developing & sustaining resources for R4H* | *60.091* |
| **(C). Producing and using research** | 0 |
| (12)National R4H Programme Action Plan Index (HRPAI) | 0 |
| (13) National Knowledge Translation Platform Index (KTPI) | 0 |
| (14) National Health Research Management Forum Index (HRMFI) | 0 |
| (15) National R4H Publications Per 100 000 population Index (PAPI)* | 6.410 |
| *Average Score for Producing and Using Research* | *1.602* |
| **(D). Financing of R4H** | 0 |
| (16)National Budget Line for R4H Index (RBLHRI) | 0 |
| (17) National Government Spending on R4H Index (RHRBI) | 0 |
| *Average Score for Financing of R4H* | *0* |
| **National Health Research Systems Average Score** | 41.58 |
|  |  |
| **Rwanda Health Research System Barometer Scores** |  |
| **Health Research System Barometer Parameters** | **Barometer Score (%)** |
| ***(A). Governance of research for health*** |  |
| (1)   National Health Research Policy Index (HRPI) | 100 |
| (2)   National Health Research Law Index (HRLI) | 100 |
| (3)   National Strategic Health Research Plan Index (SHRPI) | 100 |
| (4)   National Ethical Review Committee (ERCI) Index | 100 |
| (5)   National Health Research Priority List Index (HRPLI) | 100 |
| (6)   National Health Research Focal Point Index (HRFPI) | 100 |
| *Average score for the governance of R4H* | *100* |
| **(B). Developing and sustaining resources for R4H** | 0 |
| (7)   National Universities with Faculties of Health Sciences/Medicine (UFHSI) | 0.413 |
| (8)   National Health Research Institutes or Council (HRCI) | 100 |
| (9)   National R4H Programme (HRPRI) | 100 |
| (10) National R4H Programme Staff Density Index (RHRHRI) | 0.124 |
| (11) National NGOs R4H Index (NGOI) | 100 |
| *Average score for developing & sustaining resources for R4H* | *60.107* |
| **(C). Producing and using research** | 0 |
| (12)National R4H Programme Action Plan Index (HRPAI) | 100 |
| (13) National Knowledge Translation Platform Index (KTPI) | 100 |
| (14) National Health Research Management Forum Index (HRMFI) | 100 |
| (15) National R4H Publications Per 100 000 population Index (PAPI)* | 3.358 |
| *Average Score for Producing and Using Research* | *75.839* |
| **(D). Financing of R4H** | 0 |
| (16)National Budget Line for R4H Index (RBLHRI) | 100 |
| (17) National Government Spending on R4H Index (RHRBI) | 70.972 |
| *Average Score for Financing of R4H* | *85.486* |
| **National Health Research Systems Average Score** | **80.87** |
|  |  |
| **Sao Tome and Principe Health Research System Barometer Scores** | |
| **Health Research System Barometer Parameters** | **Barometer Score (%)** |
| ***(A). Governance of research for health*** |  |
| (1)   National Health Research Policy Index (HRPI) | 0 |
| (2)   National Health Research Law Index (HRLI) | 0 |
| (3)   National Strategic Health Research Plan Index (SHRPI) | 0 |
| (4)   National Ethical Review Committee (ERCI) Index | 100 |
| (5)   National Health Research Priority List Index (HRPLI) | 0 |
| (6)   National Health Research Focal Point Index (HRFPI) | 0 |
| *Average score for the governance of R4H* | *16.667* |
| **(B). Developing and sustaining resources for R4H** |  |
| (7)   National Universities with Faculties of Health Sciences/Medicine (UFHSI) | 0 |
| (8)   National Health Research Institutes or Council (HRCI) | 0 |
| (9)   National R4H Programme (HRPRI) | 0 |
| (10) National R4H Programme Staff Density Index (RHRHRI) | 0 |
| (11) National NGOs R4H Index (NGOI) | 0 |
| *Average score for developing & sustaining resources for R4H* | *0* |
| **(C). Producing and using research** |  |
| (12)National R4H Programme Action Plan Index (HRPAI) | 0 |
| (13) National Knowledge Translation Platform Index (KTPI) | 0 |
| (14) National Health Research Management Forum Index (HRMFI) | 0 |
| (15) National R4H Publications Per 100 000 population Index (PAPI)* | 8.228 |
| Average Score for Producing and Using Research | 2.057 |
| **(D). Financing of R4H** |  |
| (16)National Budget Line for R4H Index (RBLHRI) | 0 |
| (17) National Government Spending on R4H Index (RHRBI) | 0 |
| *Average Score for Financing of R4H* | *0* |
| **National Health Research Systems Average Score** | **6.37** |
|  |  |
| **Senegal Health Research System Barometer Scores** |  |
| **Health Research System Barometer Parameters** | **Barometer Score (%)** |
| ***(A). Governance of research for health*** |  |
| (1)   National Health Research Policy Index (HRPI) | 100 |
| (2)   National Health Research Law Index (HRLI) | 100 |
| (3)   National Strategic Health Research Plan Index (SHRPI) | 100 |
| (4)   National Ethical Review Committee (ERCI) Index | 100 |
| (5)   National Health Research Priority List Index (HRPLI) | 100 |
| (6)   National Health Research Focal Point Index (HRFPI) | 100 |
| *Average score for the governance of R4H* | *100* |
| **(B). Developing and sustaining resources for R4H** |  |
| (7)   National Universities with Faculties of Health Sciences/Medicine (UFHSI) | 1.379 |
| (8)   National Health Research Institutes or Council (HRCI) | 0 |
| (9)   National R4H Programme (HRPRI) | 100 |
| (10) National R4H Programme Staff Density Index (RHRHRI) | 0.048 |
| (11) National NGOs R4H Index (NGOI) | 100 |
| **Average score for developing & sustaining resources for R4H** | **40.286** |
| **(C). Producing and using research** |  |
| (12)National R4H Programme Action Plan Index (HRPAI) | 100 |
| (13) National Knowledge Translation Platform Index (KTPI) | 100 |
| (14) National Health Research Management Forum Index (HRMFI) | 100 |
| (15) National R4H Publications Per 100 000 population Index (PAPI)* | 10.479 |
| *Average Score for Producing and Using Research* | *77.620* |
| **(D). Financing of R4H** | 0 |
| (16)National Budget Line for R4H Index (RBLHRI) | 100 |
| (17) National Government Spending on R4H Index (RHRBI) | 0 |
| *Average Score for Financing of R4H* | *50* |
| **National Health Research Systems Average Score** | **71.29** |
|  |  |
| **Seychelles Health Research System Barometer Scores** |  |
| **Health Research System Barometer Parameters** | **Barometer Score (%)** |
| ***(A). Governance of research for health*** |  |
| (1)   National Health Research Policy Index (HRPI) | 0 |
| (2)   National Health Research Law Index (HRLI) | 0 |
| (3)   National Strategic Health Research Plan Index (SHRPI) | 0 |
| (4)   National Ethical Review Committee (ERCI) Index | 100 |
| (5)   National Health Research Priority List Index (HRPLI) | 0 |
| (6)   National Health Research Focal Point Index (HRFPI) | 0 |
| *Average score for the governance of R4H* | *16.667* |
| **(B). Developing and sustaining resources for R4H** | 0 |
| (7)   National Universities with Faculties of Health Sciences/Medicine (UFHSI) | 100 |
| (8)   National Health Research Institutes or Council (HRCI) | 0 |
| (9)   National R4H Programme (HRPRI) | 0 |
| (10) National R4H Programme Staff Density Index (RHRHRI) | 0 |
| (11) National NGOs R4H Index (NGOI) | 0 |
| *Average score for developing & sustaining resources for R4H* | *20* |
| **(C). Producing and using research** | 0 |
| (12)National R4H Programme Action Plan Index (HRPAI) | 0 |
| (13) National Knowledge Translation Platform Index (KTPI) | 0 |
| (14) National Health Research Management Forum Index (HRMFI) | 0 |
| (15) National R4H Publications Per 100 000 population Index (PAPI)* | 100 |
| *Average Score for Producing and Using Research* | *25* |
| **(D). Financing of R4H** | 0 |
| (16)National Budget Line for R4H Index (RBLHRI) | 0 |
| (17) National Government Spending on R4H Index (RHRBI) | 0 |
| *Average Score for Financing of R4H* | *0* |
| **National Health Research Systems Average Score** | 17.65 |
|  |  |
| **Sierra Leone Health Research System Barometer Scores** |  |
| ***Health Research System Barometer Parameters*** | ***Barometer Score (%)*** |
| (A). Governance of research for health |  |
| (1)   National Health Research Policy Index (HRPI) | 100 |
| (2)   National Health Research Law Index (HRLI) | 0 |
| (3)   National Strategic Health Research Plan Index (SHRPI) | 0 |
| (4)   National Ethical Review Committee (ERCI) Index | 0 |
| (5)   National Health Research Priority List Index (HRPLI) | 0 |
| (6)   National Health Research Focal Point Index (HRFPI) | 100 |
| *Average score for the governance of R4H* | *33.333* |
| ***(B). Developing and sustaining resources for R4H*** | 0 |
| (7)   National Universities with Faculties of Health Sciences/Medicine (UFHSI) | 3.226 |
| (8)   National Health Research Institutes or Council (HRCI) | 0 |
| (9)   National R4H Programme (HRPRI) | 100 |
| (10) National R4H Programme Staff Density Index (RHRHRI) | 0.065 |
| (11) National NGOs R4H Index (NGOI) | 0 |
| *Average score for developing & sustaining resources for R4H* | *20.658* |
| ***(C). Producing and using research*** | 0 |
| (12)National R4H Programme Action Plan Index (HRPAI) | 0 |
| (13) National Knowledge Translation Platform Index (KTPI) | 0 |
| (14) National Health Research Management Forum Index (HRMFI) | 0 |
| (15) National R4H Publications Per 100 000 population Index (PAPI)* | 2.708 |
| *Average Score for Producing and Using Research* | *0.677* |
| ***(D). Financing of R4H*** | 0 |
| (16)National Budget Line for R4H Index (RBLHRI) | 0 |
| (17) National Government Spending on R4H Index (RHRBI) | 0 |
| *Average Score for Financing of R4H* | *0* |
| **National Health Research Systems Average Score** | 18.00 |
|  |  |
| **South Africa Health Research System Barometer Scores** |  |
| ***Health Research System Barometer Parameters*** | ***Barometer Score (%)*** |
| (A). Governance of research for health |  |
| (1)   National Health Research Policy Index (HRPI) | 100 |
| (2)   National Health Research Law Index (HRLI) | 100 |
| (3)   National Strategic Health Research Plan Index (SHRPI) | 100 |
| (4)   National Ethical Review Committee (ERCI) Index | 100 |
| (5)   National Health Research Priority List Index (HRPLI) | 100 |
| (6)   National Health Research Focal Point Index (HRFPI) | 100 |
| *Average score for the governance of R4H* | *100* |
| ***(B). Developing and sustaining resources for R4H*** | 0 |
| (7)   National Universities with Faculties of Health Sciences/Medicine (UFHSI) | 0.753 |
| (8)   National Health Research Institutes or Council (HRCI) | 100 |
| (9)   National R4H Programme (HRPRI) | 100 |
| (10) National R4H Programme Staff Density Index (RHRHRI) | 0 |
| (11) National NGOs R4H Index (NGOI) | 100 |
| *Average score for developing & sustaining resources for R4H* | *60.151* |
| ***(C). Producing and using research*** | 0 |
| (12)National R4H Programme Action Plan Index (HRPAI) | 100 |
| (13) National Knowledge Translation Platform Index (KTPI) | 100 |
| (14) National Health Research Management Forum Index (HRMFI) | 100 |
| (15) National R4H Publications Per 100 000 population Index (PAPI)* | 40.033 |
| *Average Score for Producing and Using Research* | *85.008* |
| (D). Financing of R4H | 0 |
| (16)National Budget Line for R4H Index (RBLHRI) | 100 |
| (17) National Government Spending on R4H Index (RHRBI) | 0 |
| *Average Score for Financing of R4H* | *50* |
| **National Health Research Systems Average Score** | *78.87* |
|  |  |
| **South Sudan Health Research System Barometer Scores** |  |
| **Health Research System Barometer Parameters** | **Barometer Score (%)** |
| ***(A). Governance of research for health*** |  |
| (1)   National Health Research Policy Index (HRPI) | 0 |
| (2)   National Health Research Law Index (HRLI) | 0 |
| (3)   National Strategic Health Research Plan Index (SHRPI) | 0 |
| (4)   National Ethical Review Committee (ERCI) Index | 100 |
| (5)   National Health Research Priority List Index (HRPLI) | 0 |
| (6)   National Health Research Focal Point Index (HRFPI) | 100 |
| *Average score for the governance of R4H* | *33.333* |
| **(B). Developing and sustaining resources for R4H** | 0 |
| (7)   National Universities with Faculties of Health Sciences/Medicine (UFHSI) | 0.427 |
| (8)   National Health Research Institutes or Council (HRCI) | 0 |
| (9)   National R4H Programme (HRPRI) | 0 |
| (10) National R4H Programme Staff Density Index (RHRHRI) | 0 |
| (11) National NGOs R4H Index (NGOI) | 0 |
| *Average score for developing & sustaining resources for R4H* | *0.085* |
| **(C). Producing and using research** | 0 |
| (12)National R4H Programme Action Plan Index (HRPAI) | 0 |
| (13) National Knowledge Translation Platform Index (KTPI) | 0 |
| (14) National Health Research Management Forum Index (HRMFI) | 0 |
| (15) National R4H Publications Per 100 000 population Index (PAPI)* | 0 |
| *Average Score for Producing and Using Research* | *0* |
| **(D). Financing of R4H** | 0 |
| (16)National Budget Line for R4H Index (RBLHRI) | 0 |
| (17) National Government Spending on R4H Index (RHRBI) | 0 |
| *Average Score for Financing of R4H* | *0* |
| **National Health Research Systems Average Score** | **11.79** |
|  |  |
| **Swaziland Health Research System Barometer Scores** |  |
| **Health Research System Barometer Parameters** | **Barometer Score (%)** |
| ***(A). Governance of research for health*** |  |
| (1)   National Health Research Policy Index (HRPI) | 100 |
| (2)   National Health Research Law Index (HRLI) | 0 |
| (3)   National Strategic Health Research Plan Index (SHRPI) | 100 |
| (4)   National Ethical Review Committee (ERCI) Index | 100 |
| (5)   National Health Research Priority List Index (HRPLI) | 0 |
| (6)   National Health Research Focal Point Index (HRFPI) | 100 |
| *Average score for the governance of R4H* | *66.667* |
| **(B). Developing and sustaining resources for R4H** | 0 |
| (7)   National Universities with Faculties of Health Sciences/Medicine (UFHSI) | 11.538 |
| (8)   National Health Research Institutes or Council (HRCI) | 0 |
| (9)   National R4H Programme (HRPRI) | 100 |
| (10) National R4H Programme Staff Density Index (RHRHRI) | 0.231 |
| (11) National NGOs R4H Index (NGOI) | 100 |
| *Average score for developing & sustaining resources for R4H* | *42.354* |
| **(C). Producing and using research** | 0 |
| (12)National R4H Programme Action Plan Index (HRPAI) | 100 |
| (13) National Knowledge Translation Platform Index (KTPI) | 100 |
| (14) National Health Research Management Forum Index (HRMFI) | 100 |
| (15) National R4H Publications Per 100 000 population Index (PAPI)* | 9.236 |
| *Average Score for Producing and Using Research* | *77.309* |
| **(D). Financing of R4H** | 0 |
| (16)National Budget Line for R4H Index (RBLHRI) | 0 |
| (17) National Government Spending on R4H Index (RHRBI) | 0 |
| *Average Score for Financing of R4H* | *0* |
| **National Health Research Systems Average Score** | **54.18** |
|  |  |
| **Tanzania Health Research System Barometer Scores** |  |
| **Health Research System Barometer Parameters** | **Barometer Score (%)** |
| *(A). Governance of research for health* |  |
| (1)   National Health Research Policy Index (HRPI) | 100 |
| (2)   National Health Research Law Index (HRLI) | 100 |
| (3)   National Strategic Health Research Plan Index (SHRPI) | 100 |
| (4)   National Ethical Review Committee (ERCI) Index | 100 |
| (5)   National Health Research Priority List Index (HRPLI) | 100 |
| (6)   National Health Research Focal Point Index (HRFPI) | 100 |
| *Average score for the governance of R4H* | *100* |
| **(B). Developing and sustaining resources for R4H** |  |
| (7)   National Universities with Faculties of Health Sciences/Medicine (UFHSI) | 0.197 |
| (8)   National Health Research Institutes or Council (HRCI) | 100 |
| (9)   National R4H Programme (HRPRI) | 100 |
| (10) National R4H Programme Staff Density Index (RHRHRI) | 0.002 |
| (11) National NGOs R4H Index (NGOI) | 100 |
| *Average score for developing & sustaining resources for R4H* | *60.040* |
| **(C). Producing and using research** | 0 |
| (12)National R4H Programme Action Plan Index (HRPAI) | 0 |
| (13) National Knowledge Translation Platform Index (KTPI) | 100 |
| (14) National Health Research Management Forum Index (HRMFI) | 100 |
| (15) National R4H Publications Per 100 000 population Index (PAPI)* | 5.458 |
| *Average Score for Producing and Using Research* | *51.364* |
| **(D). Financing of R4H** | 0 |
| (16)National Budget Line for R4H Index (RBLHRI) | 100 |
| (17) National Government Spending on R4H Index (RHRBI) | 100 |
| *Average Score for Financing of R4H* | *100* |
| **National Health Research Systems Average Score** | **76.80** |
|  |  |
| **Togo Health Research System Barometer Scores** |  |
| **Health Research System Barometer Parameters** | **Barometer Score (%)** |
| ***(A). Governance of research for health*** |  |
| (1)   National Health Research Policy Index (HRPI) | 0 |
| (2)   National Health Research Law Index (HRLI) | 100 |
| (3)   National Strategic Health Research Plan Index (SHRPI) | 0 |
| (4)   National Ethical Review Committee (ERCI) Index | 100 |
| (5)   National Health Research Priority List Index (HRPLI) | 0 |
| (6)   National Health Research Focal Point Index (HRFPI) | 100 |
| *Average score for the governance of R4H* | *50* |
| **(B). Developing and sustaining resources for R4H** | 0 |
| (7)   National Universities with Faculties of Health Sciences/Medicine (UFHSI) | 0.714 |
| (8)   National Health Research Institutes or Council (HRCI) | 0 |
| (9)   National R4H Programme (HRPRI) | 0 |
| (10) National R4H Programme Staff Density Index (RHRHRI) | 0 |
| (11) National NGOs R4H Index (NGOI) | 0 |
| *Average score for developing & sustaining resources for R4H* | *0.143* |
| **(C). Producing and using research** | 0 |
| (12)National R4H Programme Action Plan Index (HRPAI) | 0 |
| (13) National Knowledge Translation Platform Index (KTPI) | 0 |
| (14) National Health Research Management Forum Index (HRMFI) | 0 |
| (15) National R4H Publications Per 100 000 population Index (PAPI)* | 5.346 |
| *Average Score for Producing and Using Research* | *1.337* |
| **(D). Financing of R4H** | 0 |
| (16)National Budget Line for R4H Index (RBLHRI) | 0 |
| (17) National Government Spending on R4H Index (RHRBI) | 0 |
| *Average Score for Financing of R4H* | *0* |
| **National Health Research Systems Average Score** | 18.00 |
|  |  |
| **Uganda Health Research System Barometer Scores** |  |
| **Health Research System Barometer Parameters** | **Barometer Score (%)** |
| ***(A). Governance of research for health*** |  |
| (1)   National Health Research Policy Index (HRPI) | 100 |
| (2)   National Health Research Law Index (HRLI) | 100 |
| (3)   National Strategic Health Research Plan Index (SHRPI) | 100 |
| (4)   National Ethical Review Committee (ERCI) Index | 100 |
| (5)   National Health Research Priority List Index (HRPLI) | 100 |
| (6)   National Health Research Focal Point Index (HRFPI) | 100 |
| *Average score for the governance of R4H* | *100* |
| **(B). Developing and sustaining resources for R4H** | 0 |
| (7)   National Universities with Faculties of Health Sciences/Medicine (UFHSI) | 0.387 |
| (8)   National Health Research Institutes or Council (HRCI) | 0 |
| (9)   National R4H Programme (HRPRI) | 100 |
| (10) National R4H Programme Staff Density Index (RHRHRI) | 0 |
| (11) National NGOs R4H Index (NGOI) | 100 |
| *Average score for developing & sustaining resources for R4H* | *40.077* |
| **(C). Producing and using research** | 0 |
| (12)National R4H Programme Action Plan Index (HRPAI) | 100 |
| (13) National Knowledge Translation Platform Index (KTPI) | 100 |
| (14) National Health Research Management Forum Index (HRMFI) | 100 |
| (15) National R4H Publications Per 100 000 population Index (PAPI)* | 8.025 |
| *Average Score for Producing and Using Research* | *77.006* |
| **(D). Financing of R4H** | 0 |
| (16)National Budget Line for R4H Index (RBLHRI) | 100 |
| (17) National Government Spending on R4H Index (RHRBI) | 14.158 |
| *Average Score for Financing of R4H* | *57.079* |
| **National Health Research Systems Average Score** | **71.92** |
|  |  |
| **Zambia Health Research System Barometer Scores** |  |
| **Health Research System Barometer Parameters** | **Barometer Score (%)** |
| **(A). Governance of research for health** |  |
| (1)   National Health Research Policy Index (HRPI) | 0 |
| (2)   National Health Research Law Index (HRLI) | 100 |
| (3)   National Strategic Health Research Plan Index (SHRPI) | 100 |
| (4)   National Ethical Review Committee (ERCI) Index | 100 |
| (5)   National Health Research Priority List Index (HRPLI) | 100 |
| (6)   National Health Research Focal Point Index (HRFPI) | 100 |
| *Average score for the governance of R4H* | 83.333 |
| **(B). Developing and sustaining resources for R4H** | 0 |
| (7)   National Universities with Faculties of Health Sciences/Medicine (UFHSI) | 0.667 |
| (8)   National Health Research Institutes or Council (HRCI) | 0 |
| (9)   National R4H Programme (HRPRI) | 100 |
| (10) National R4H Programme Staff Density Index (RHRHRI) | 0 |
| (11) National NGOs R4H Index (NGOI) | 100 |
| *Average score for developing & sustaining resources for R4H* | 40.133 |
| **(C). Producing and using research** | 0 |
| (12)National R4H Programme Action Plan Index (HRPAI) | 100 |
| (13) National Knowledge Translation Platform Index (KTPI) | 100 |
| (14) National Health Research Management Forum Index (HRMFI) | 100 |
| (15) National R4H Publications Per 100 000 population Index (PAPI)* | 6.359 |
| *Average Score for Producing and Using Research* | 76.590 |
| **(D). Financing of R4H** | 0 |
| (16)National Budget Line for R4H Index (RBLHRI) | 100 |
| (17) National Government Spending on R4H Index (RHRBI) | 1.063 |
| *Average Score for Financing of R4H* | 50.532 |
| **National Health Research Systems Average Score** | 65.18 |
|  |  |
| **Zimbabwe Health Research System Barometer Scores** |  |
| **Health Research System Barometer Parameters** | **Barometer Score (%)** |
| ***(A). Governance of research for health*** |  |
| (1)   National Health Research Policy Index (HRPI) | 0 |
| (2)   National Health Research Law Index (HRLI) | 100 |
| (3)   National Strategic Health Research Plan Index (SHRPI) | 0 |
| (4)   National Ethical Review Committee (ERCI) Index | 100 |
| (5)   National Health Research Priority List Index (HRPLI) | 100 |
| (6)   National Health Research Focal Point Index (HRFPI) | 100 |
| *Average score for the governance of R4H* | 66.667 |
| **(B). Developing and sustaining resources for R4H** | 0 |
| (7)   National Universities with Faculties of Health Sciences/Medicine (UFHSI) | 1.712 |
| (8)   National Health Research Institutes or Council (HRCI) | 100 |
| (9)   National R4H Programme (HRPRI) | 100 |
| (10) National R4H Programme Staff Density Index (RHRHRI) | 0.473 |
| (11) National NGOs R4H Index (NGOI) | 100 |
| *Average score for developing & sustaining resources for R4H* | 60.437 |
| (C). Producing and using research | 0 |
| (12)National R4H Programme Action Plan Index (HRPAI) | 100 |
| (13) National Knowledge Translation Platform Index (KTPI) | 100 |
| (14) National Health Research Management Forum Index (HRMFI) | 0 |
| (15) National R4H Publications Per 100 000 population Index (PAPI)* | 7.882 |
| *Average Score for Producing and Using Research* | 51.971 |
| **(D). Financing of R4H** | 0 |
| (16)National Budget Line for R4H Index (RBLHRI) | 100 |
| (17) National Government Spending on R4H Index (RHRBI) | 94.228 |
| *Average Score for Financing of R4H* | 97.114 |
| **National Health Research Systems Average Score** | 64.96 |
